# Effectiveness of Non-Pharmacological Interventions for Overweight or Obese Infertile Women: A Systematic Review and Meta-Analysis

**DOI:** 10.3390/ijerph17207438

**Published:** 2020-10-13

**Authors:** Seo Yun Kim, Eun-Sun Park, Hae Won Kim

**Affiliations:** 1 College of Nursing, Seoul National University, Seoul 03080, Korea; seo3915@snu.ac.kr; 2 Medical Library, Seoul National University, Seoul 03080, Korea; peunsun@snu.ac.kr; 3 Research Institute of Nursing Science, College of Nursing, Seoul National University, Seoul 03080, Korea

**Keywords:** obesity, preconception care, healthy lifestyle, weight loss, meta-analysis

## Abstract

Obesity is a well-known risk factor for infertility, and nonpharmacological treatments are recommended as effective and safe, but evidence is still lacking on whether nonpharmacological interventions improve fertility in overweight or obese women. The aim of this study was to systematically assess the current evidence in the literature and to evaluate the impact of nonpharmacological interventions on improving pregnancy-related outcomes in overweight or obese infertile women. Seven databases were searched for randomized controlled trials (RCTs) of nonpharmacological interventions for infertile women with overweight or obesity through August 16, 2019 with no language restriction. A meta-analysis was conducted of the primary outcomes. A total of 21 RCTs were selected and systematically reviewed. Compared to the control group, nonpharmacological interventions significantly increased the pregnancy rate (relative risk (RR), 1.37; 95% CI, 1.04–1.81; *p* = 0.03; I^2^ = 58%; nine RCTs) and the natural conception rate (RR, 2.17, 95% CI, 1.41–3.34; *p* = 0.0004; I^2^ = 19%, five RCTs). However, they had no significant effect on the live birth rate (RR, 1.36, 95% CI, 0.94–1.95; *p*=0.10, I^2^ = 65%, eight RCTs) and increased the risk of miscarriage (RR: 1.57, 95% CI, 1.05–2.36; *p* = 0.03; I^2^ = 0%). Therefore, nonpharmacological interventions could have a positive effect on the pregnancy and natural conception rates, whereas it is unclear whether they improve the live birth rate. Further research is needed to demonstrate the integrated effects of nonpharmacological interventions involving psychological outcomes, as well as pregnancy-related outcomes.

## 1. Introduction

Infertility is a major health concern that affects 8–12% of couples of childbearing age who are trying to conceive worldwide [1]. Overweight and obesity are well-known risk factors for infertility that can increase the likelihood of maternal and fetal/neonatal adverse outcomes (e.g., gestational hypertension, pre-eclampsia, perinatal depression, fetal defects, and perinatal mortality) [2,3].

The mechanisms through which obesity contributes to infertility include hormonal changes, menstrual disorders, and ovulatory disorders [4,5]. The majority of women with ovulatory disorders have polycystic ovary syndrome (PCOS), and a significant proportion of women with polycystic ovary symptom (PCOS) are obese [6].

With the increasing prevalence of infertility in obese women, it is becoming increasingly common for women to seek assisted reproductive treatment in order to become pregnant [7]. Overweight and obese women have poor reproductive outcomes for assisted conception techniques such as ovulation induction and in vitro fertilization/intracytoplasmic sperm injection [8,9,10]. However, weight reduction improves reproductive outcomes in these patients [8,11,12,13].

Pharmacological treatments for obesity and bariatric surgery are limited as options for overweight or obese infertile women, because the safety of these treatment modalities in terms of pregnancy outcomes has not been thoroughly investigated [14,15,16]. Due to the potential risks associated with surgery or weight loss medications, health organizations have recommended that overweight or obese infertile women pursue lifestyle changes, including dietary changes and physical activity, as nonpharmacological weight loss interventions [17]. Recent international guidelines strongly support the importance of preconception lifestyle interventions in an interdisciplinary setting to promote healthy lifestyles and to sustain weight loss for obese women [14,18]. In addition, knowledge is emerging regarding the effects of supplements and complementary therapy (herbal medicine and acupuncture), but the evidence of the overall effects of these interventions is incomplete. 

Although nonpharmacological treatment is considered to be an effective and safe method for managing obesity in infertile women, an ongoing challenge is that it is unclear to what extent nonpharmacological methods of obesity treatment improve fertility outcomes. Therefore, it is imperative to clearly summarize the current evidence on nonpharmacological interventions and their effects on fertility outcomes in overweight and obese infertile women. The results of this study will yield insights into future directions in education and counseling by health professionals, including nurses, by providing information on the degree to which the pregnancy outcomes are likely to be improved by nonpharmacological treatments in overweight or obese women seeking pregnancy. 

The aim of this review was to systematically assess the current literature and to evaluate the impact of nonpharmacological interventions in overweight and obese infertile women. Specifically, the goals of this study were (1) to identify the characteristics of studies of nonpharmacological interventions and (2) to synthesize data on the effects of different types of nonpharmacological interventions on pregnancy and birth outcomes (pregnancy, natural conception, live birth, and miscarriage rates). 

## 2. Materials and Methods 

This study is a systematic literature review and meta-analysis of nonpharmacological intervention studies conducted on overweight and obese infertile women. This systematic literature review followed the Preferred Reporting Items for Systematic Reviews and Meta-Analyses (PRISMA) guidelines [19]. No protocol was registered. 

### 2.1. Selection Criteria 

#### 2.1.1. Inclusion Criteria

The key questions for the systematic literature review were based on the Populations, Intervention, Comparison, Outcome, and Study Design (PICOSD) framework as follows: (1) The subjects were overweight or obese adult women (with a body mass index (BMI) of at least 25 kg/m2) who were diagnosed with infertility, were undergoing or scheduled to undergo infertility procedures, or were referred to infertility clinics and were actively trying to conceive (P). (2) The nonpharmacological interventions included diet; exercise; weight control programs; alternative complementary therapies (e.g., yoga, acupuncture, aromatherapy, relaxation, meditation, mindfulness, and hypnosis); psychosocial interventions; cognitive behavioral programs; nursing interventions; and combinations thereof. The interventions could be combined with pharmacological therapy, but the focus was on including studies that verified the effectiveness of nonpharmacological interventions or interventions with dietary supplements (I). (3) The comparison was any group that did not receive a nonpharmacological intervention (usual care, no treatment, pharmacological treatment only, or placebo) (C). (4) The chosen outcomes were those that have been consistently reported in the literature and were analyzed as primary or secondary outcomes according to their frequency, validity, and clinical significance. Our primary outcomes included pregnancy- and birth-related outcomes (pregnancy, natural conception, live birth, and miscarriage rates), and the secondary outcomes included anthropometrics (changes in weight, BMI, and waist circumference); fertility outcomes (ovulation rate and menstrual cycle improvements); reproductive hormone levels; metabolic hormone levels; psychological outcomes; cognitive behavioral outcomes; and adverse outcomes (O). (5) The review included all prospective randomized controlled trials (S). We included conference abstract/abstract only or protocol studies if they reported results.

#### 2.1.2. Exclusion Criteria 

Studies were excluded if they involved adolescents or women who were not currently trying to conceive. Studies that only analyzed pharmacological interventions or bariatric surgery were excluded. Studies were limited to those with results or tables of results being available in English, and nonexperimental studies, such as theoretical studies or editorial comments, protocol-only studies, and review papers were excluded.

### 2.2. Search Strategy and Data Extraction

#### 2.2.1. Search Strategy

The search strategy was developed and conducted by a medical librarian with extensive experience in systematic reviews with input from the study authors. The search was conducted on 16 August 2019 using the following electronic databases: Ovid MEDLINE, EMbase, Cochrane Library, CINAHL, Web of Science, PsycINFO, and a Korean domestic database (KoreaMed). The search strategy was as follows: in the first step, a preliminary search was conducted based on a combination of terms relating to overweight/obesity and infertility (P) and nonpharmacologic interventions (I), and a comprehensive search strategy was established after reviewing the abstracts and indexing the terms of the retrieved studies. In the second step, the Medical Subject Headings (MeSH) and Emtree were identified using the selected search terms. In the third step, a search strategy was established by appropriately utilizing Boolean operators (AND, OR, adj). The search keywords were created by combining (P), (I), and (SD) terms using the AND operator and filtered by abstract and title. The search terms used were: (“infertility*” OR “in vitro fertilization” OR “reproduction techniques” “in vitro fertilization (IVF)” OR “intrauterine insemination (IUI)”) AND (obesity* OR obese* OR overweight*) AND (“lifestyle” OR “weight reduction” OR “nutrition” OR “diet” and “cognitive behavioral therapy” OR “counseling” OR “complementary therapy” OR “exercise” and “social support” OR “nursing care”) AND (“Randomized Controlled Trial” OR “Controlled Clinical Trial”). The complete search strategy used in Ovid MEDLINE is described in Supplementary File S1. Additional studies were also searched on Google Scholar (https://scholar.google.com) through a manual review of the reference lists of the identified studies, and ClinicalTrials.gov (https://clinicaltrials.gov) was screened to identify protocol studies or ongoing studies that have reported results. No restriction was placed on language and publication year. 

#### 2.2.2. Selection of Studies

Two reviewers (H.K. and S.K.) independently screened the titles and abstracts of all studies obtained to evaluate their relevance for inclusion (the consistency of the two reviewers was 96.6%), and the full text of all relevant studies was retrieved for a further detailed evaluation (the consistency was 94.1%). Additional studies were identified by examining the reference lists of the full-text papers that were closely scrutinized for eligibility. Published abstracts or conference proceedings were also screened to determine whether they met the inclusion criteria. Any disagreement about inclusion was resolved by discussion. When more than one publication presented results from the same study population, we included the results of the most recent or complete version. 

#### 2.2.3. Data Extraction

Data were extracted by one reviewer (S.K.) using a data extraction form and checked by a second reviewer (H.K). Discrepancies between the two were resolved by consensus. General characteristics of the study (author, country, year of publication, and study design); characteristics of the study participants (mean age, mean BMI, duration of infertility, and sample size); type and description of the intervention; health professional involvement; the setting, format, duration, and number of sessions; the frequency of contacts; the follow-up period; the attrition rate; and the main outcome variables were extracted from each included study. 

### 2.3. Risk of Bias Assessment

The methodological quality of each included study was evaluated using the Cochrane risk of bias assessment (RoB) tool [20]. The risk of bias was evaluated for seven domains: random sequence generation, allocation concealment, blinding of participants and personnel, blinding of the outcome assessment, incomplete outcome data, selective reporting, and other bias. In this study, other sources of bias included baseline imbalances between groups and potential confounding factors. Each study was evaluated as having a low, high, or unclear risk of bias. The risk of bias assessment was independently conducted by two researchers (H.K. and S.K.; the consistency of the two reviewers was 90.5%), and in case of disagreement, the researchers jointly reviewed the full text to reach a consensus.

### 2.4. Statistical Analysis

The general characteristics of the studies were presented as frequencies and percentages, and the outcome variables were presented as frequencies. The meta-analysis was performed using RevMan software version 5.3 (The Cochrane Collaboration, Software Update, Oxford, UK) when a quantitative synthesis was possible for the primary outcomes from the selected studies. A random-effects model was used for analysis, because heterogeneity was considered to be present among the selected studies regarding factors such as the research methods, settings, subjects, and clinical diversity. A meta-analysis was performed when the results for a certain variable were reported by at least three studies in a format suitable for analysis, while the threshold was at least two studies in the subgroup analysis by intervention. The effect size was presented in terms of risk ratios (RRs) and mean differences with 95% CIs between groups, as each of the studies reported binary variables. The summary risk ratios (RRs) were calculated using the Mantel-Haenszel (M-H) method. Heterogeneity was examined based on the common part of the CIs, the effect was estimated by a visual inspection or forest plot, and heterogeneity was quantitatively evaluated using the Higgins I^2^ statistic (with I^2^ values of 25%, 50%, and 75%, respectively, indicating low, moderate, and high heterogeneity) [21]. To assess the publication bias, the funnel plot was evaluated by visual inspection, and the statistical significance of the asymmetry was confirmed with the Egger’s linear regression test [22] and Begg and Mazumdar’s rank correlation test [23] in the meta-analysis.

## 3. Results

### 3.1. Selected Studies

The initial electronic database search resulted in 6046 articles (386 in Ovid MEDLINE, 1614 in EMbase, 3802 in the Cochrane Library, 152 in CINAHL, 58 in the Web of Science, 5 in PsycINFO, and 29 in KoreaMed) related to nonpharmacological interventions for overweight/obesity or infertility. After the removal of duplicates (*n* = 861), 5185 articles were screened to determine whether their titles and abstracts met the inclusion criteria. After the removal of entries with an unsuitable title or abstract (*n* = 5018), the full texts of 167 studies were assessed for eligibility. A further 134 papers were removed due to the following reasons: not having a relevant study population (*n* = 80) that was ineligible for infertility criteria (*n* = 59), ineligible for infertility criteria or obesity/overweight criteria (*n* = 9), ineligible for obesity/overweight criteria (*n* = 7) and related to the infertility of males (*n* = 5), not conducing a nonpharmacological intervention (*n* = 1), not being experimental studies or randomized controlled trials (RCTs) (*n* = 15), being duplicates that were excluded by hands (*n* = 24), not presenting results or tables of results in English (*n* = 7), and presenting only a study protocol (*n* = 7). Thirteen studies excluded were during further data extraction, because they shared the same study populations; these were follow-up studies (*n* = 3), secondary analysis studies (*n* = 4), a subgroup analysis study (*n* = 1), a cost-effectiveness study (*n* = 1), and conference abstract/abstract-only studies with same population (*n* = 4). The detail information of the reasons for the exclusion of all studies that were excluded after a full-text review is listed in Supplementary File S2.

**Figure 1:**
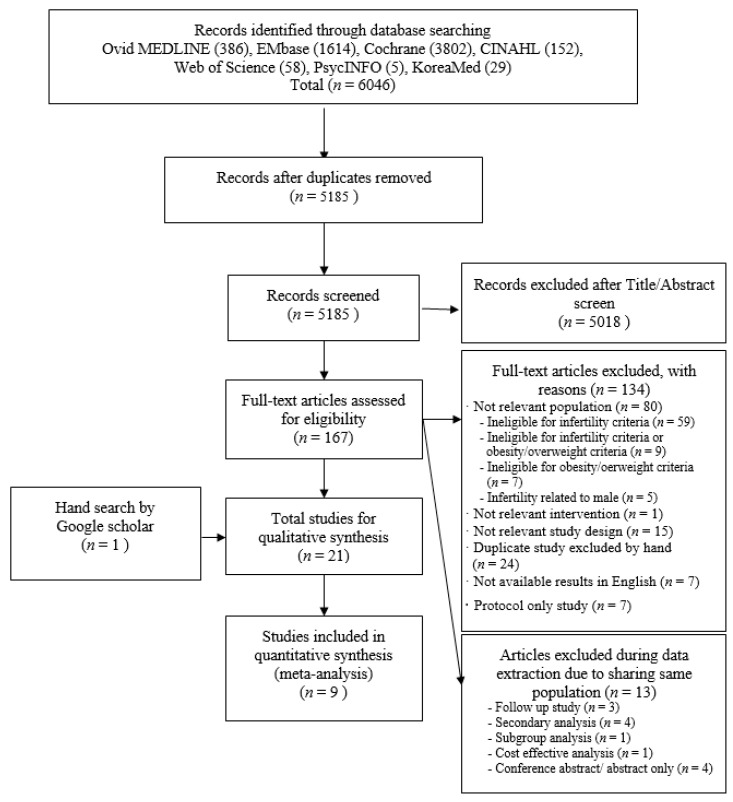
Preferred Reporting Items for Systematic Reviews and Meta-Analyses (PRISMA) flow diagram for the process of selecting included studies in the systematic review.

An additional article was included from the hand search of Google Scholar. We identified that two related studies were in progress through an additional search of ClinicalTrials.gov, but these were not included in our study, because the results were not presented. Therefore, no additional studies were included through ClinicalTrials.gov. Finally, 21 papers relevant for this systematic review were identified. Figure 1 presents the details of the selection process and the included studies in a PRISMA flow diagram. 

### 3.2. Risk of Bias Assessment

**Figure 2:**
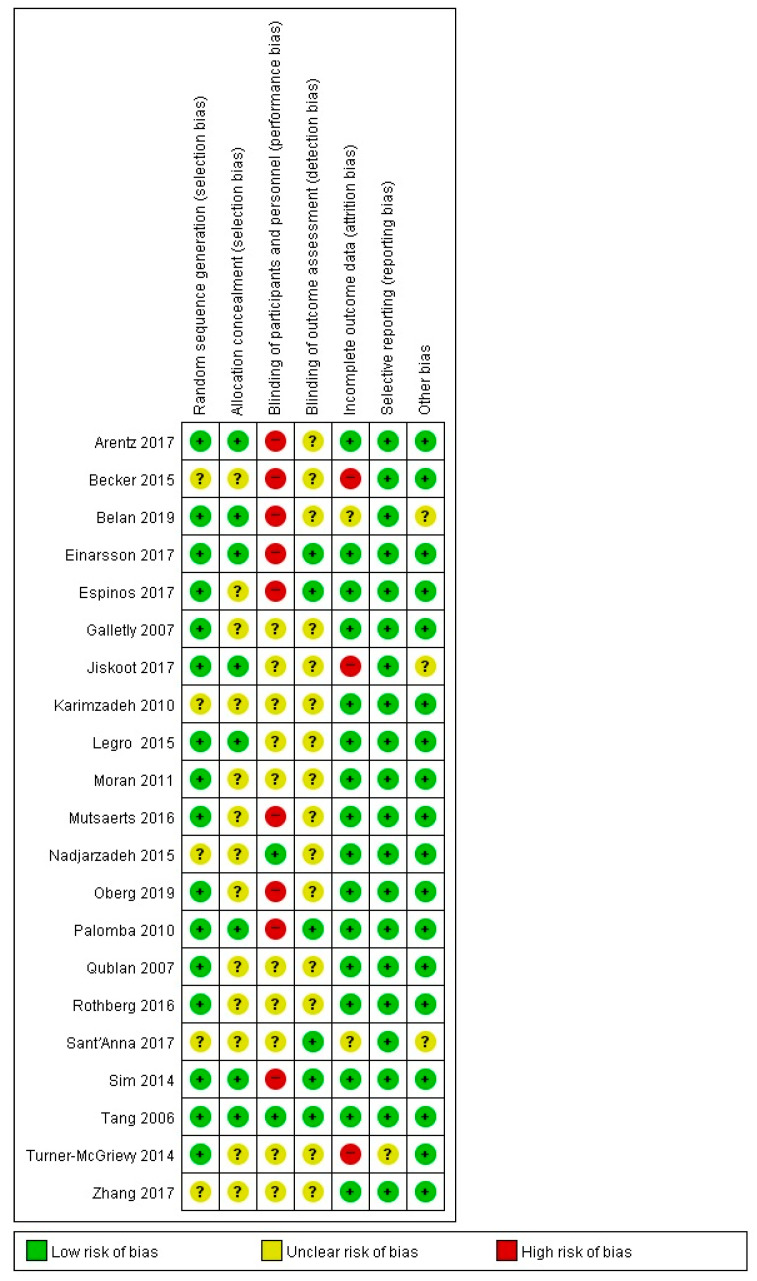
Risk of bias graph.

**Figure 3:**
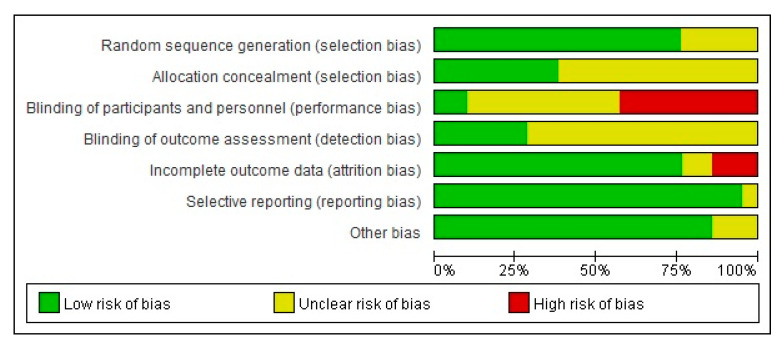
Risk of bias summary.

More than 75% of the studies had a low risk of bias for the assessment of randomized allocation to the experimental and control groups at the study design stage. The risk of bias was low for about 38% of the studies in terms of allocation concealment. Only 10% of studies had a low risk of bias in terms of blinding of the participants and researchers, while the other studies had uncertain or higher risks of bias. More than 25% of the studies had a low risk of bias for the blinding of result assessments, while the others had uncertain outcomes. The attrition bias was low in more than 75% of the studies and high in 14% of the studies. The risk of bias due to selective reporting was low in more than 95% of the studies, and more than 85% of the studies had a low risk of bias in other areas (Figure 2 and Figure 3).

### 3.3. Characteristics of the Included Studies

#### 3.3.1. General Characteristics of the Selected Studies

Table 1 and Table 2 present the characteristics of the 21 included studies by year, country, and total number of participants. The 21 included studies were conducted between 2005 and 2019, and 13 (61.9%) of them were conducted between 2015 and 2019. The studies were conducted in 14 countries, and Australia was the country where the largest number of studies (*n* = 4; 19%) was conducted. The number of participants ranged from five to 284 per group, and the plurality (*n* = 8; 38.1%) had five to 29 participants. The average age of the participants was 30 years, 10 (47.6%) studies had both experimental and control groups, and the average BMI was between 30.0 kg/m2 and 34.9 kg/m2 in eight studies (38.1%). The primary reason for infertility was PCOS-only in more than half of the studies (*n* = 12; 57.1%) (Table 1 and Table 2).

#### 3.3.2. Characteristics of Nonpharmacological Interventions

Five types of nonpharmacological interventions were provided for overweight or obese women affected by infertility: dietary interventions (*n* = 5; 23.8%); dietary and exercise-based interventions (*n* = 4; 19%); diet, exercise, and behavioral modification interventions (*n* = 5; 23.8%); lifestyle interventions combined with medication (*n* = 5; 23.8%); and supplementations (*n* = 2; 9.5%). The providers of the interventions were dietitians (*n* = 13; 37.1%), nurses or midwives (*n* = 4; 11.4%), and physicians (*n* = 4; 11.4%). Interventions were mostly provided in hospital or fertility clinic settings (*n* = 16; 76.2%). Most of the interventions were in-person *(n* = 20, 69%) and private (*n* = 9, 31%). The durations of the interventions ranged from seven and a half weeks to 18 months, with most (*n* = 10, 66.7%) provided from 13 weeks to six months. The post-intervention follow-up periods were between six weeks and 53 months, with six studies (28.6%) including seven to 12 months of follow-up (Table 2). 

#### 3.3.3. Characteristics of Outcome Variables 

The outcome variables of the interventions were divided into eight categories: anthropometrics, pregnancy and birth outcomes, fertility-related outcomes, reproductive hormone levels, metabolic hormone levels, psychological outcomes, cognitive behavioral outcomes, and adverse outcomes. The outcome variables of each category are listed in Table 3. The main outcome variables of each category were body weight (*n* = 18) and BMI (*n* = 16) for anthropometrics; pregnancy (*n* = 17), natural conception (*n* = 6), live birth (*n* = 11), and miscarriage (n = 9) rates for pregnancy and birth outcomes; ovulation-related variables (ovulation rate, *n* = 5 and ovulation detection, *n* = 2) and menstrual cycle improvement-related variables (regularity, *n* = 5 and frequency, *n* = 2) for fertility related-outcomes; gonadotropin hormone levels (*n* = 16), including the luteinizing hormone and follicle-stimulating hormone, as well as androgen hormones (*n* = 20) such as testosterone and androstenedione for reproductive hormones; glucose levels (*n* = 9), insulin levels (*n* = 19), and lipid profiles (*n* = 14) for metabolic outcomes; quality of life (*n* = 5) and depression *(n* = 4) for psychological outcomes; and dietary intake (*n* = 3), physical activity (*n* = 3), and self-esteem (*n* = 2) for cognitive behavioral outcomes. Several variables were measured in the adverse outcome category depending on the timing of the pregnancy (ranging from preconception to postpartum) (*n* = 11) (Table 3). 

### 3.4. Effects of the Nonpharmacological Interventions

For pregnancy and birth outcomes, which were the primary outcomes of the 21 studies, the effect sizes were measured for the pregnancy rate, natural conception rate, live birth rate, and miscarriage rate. Subgroup effects were analyzed depending on whether the intervention type was (a) a dietary intervention vs. an usual diet, no treatment, or medication; (b) a dietary and exercise-based intervention vs. an usual diet, no treatment, or medication; and (c) and diet, exercise, and behavioral modification counseling vs. the usual care.

#### 3.4.1. Pregnancy Rate 

**Figure 4:**
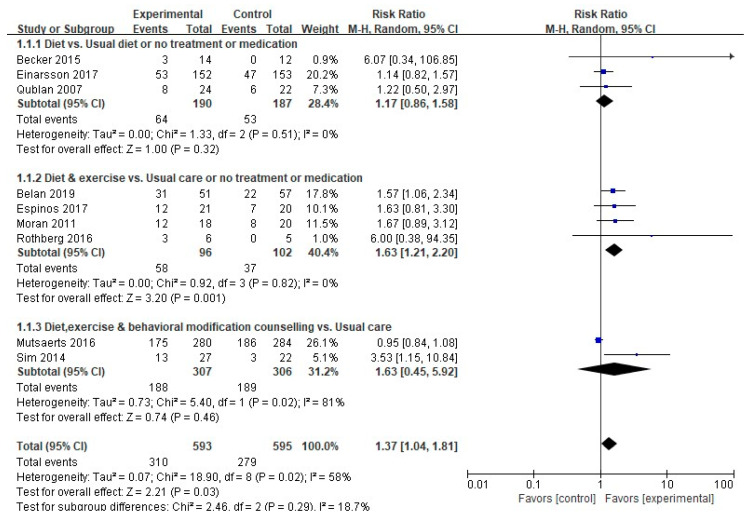
Forest plot of non-pharmacological intervention on pregnancy rate. M-H, the Mantel-Haenszel method; Random, random effects model; CI, confidence interval.

A meta-analysis was performed on nine out of the 17 studies that measured pregnancy outcomes as a primary variable, excluding eight studies with nonpharmacological interventions in the control group (*n* = 2), a combined pharmacological intervention (*n* = 5), or no comparisons between the experimental and control groups (*n* = 1). The pregnancy rate was meaningfully higher in the experimental group than in the control group (310/593 (52.3%) vs. 279/595 (46.9%); RR, 1.37; 95% CI, 1.04–1.81; *p* = 0.03). A subgroup analysis was performed, because there was medium heterogeneity (Higgins I^2^ = 58%) (Figure 4). 

In the four studies that carried out interventions combining diet and exercise, the interventions demonstrated meaningful differences in boosting pregnancy rates compared to the usual care or no treatments (58/96 (60.4%) vs. 37/102 (36.3%); RR, 1.63; 95% CI, 1.21–2.20; *p* = 0.001), with no heterogeneity (Higgins I^2^ = 0%). 

#### 3.4.2. Natural Conception Rate

**Figure 5:**
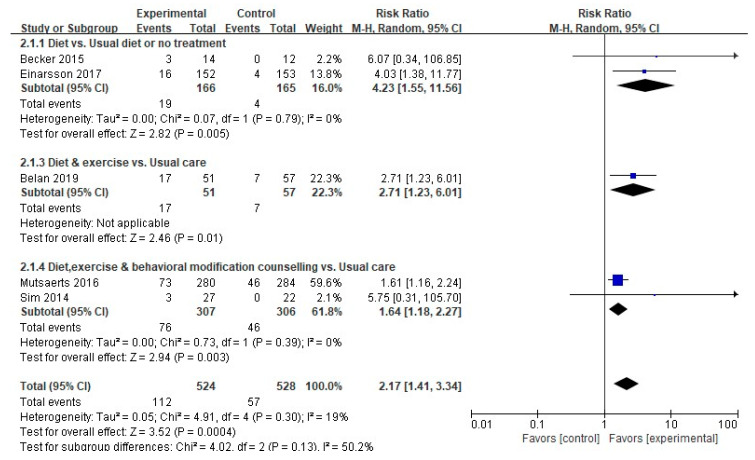
Forest plot of nonpharmacological interventions on the natural conception rate. M-H, the Mantel-Haenszel method; Random, random effects model; CI, confidence interval.

A meta-analysis was performed on five of the six studies that measured the natural conception rate as a primary outcome, excluding one that did not provide a comparison between the experimental and control groups (*n* = 1). The experimental group showed a meaningfully higher natural conception rate compared to the control group (112/524 (21.4%) vs. 57/528 (10.8%); RR, 2.17; 95% CI, 1.41-3.34; *p* = 0.0004), with low heterogeneity (Higgins I^2^ = 19%) (Figure 5). The subgroup analysis found that the two studies with dietary-only interventions demonstrated a meaningful difference in the natural conception rate between the experimental and control groups (19/166 (11.4%) vs. 4/165 (2.4%); RR, 4.23; 95% CI, 1.5-11.56; *p* = 0.005), with no heterogeneity (Higgins I^2^ = 0%). 

The natural conception rate was meaningfully different between the experimental and control groups in the two studies that involved diet, exercise, and behavioral modification counseling (76/307 (23.8%) vs. 46/306 (15.0%); RR, 1.64; 95% CI, 1.18–2.27; *p* = 0.03), demonstrating no heterogeneity (Higgins I^2^ = 0%).

#### 3.4.3. Live Birth Rate

**Figure 6:**
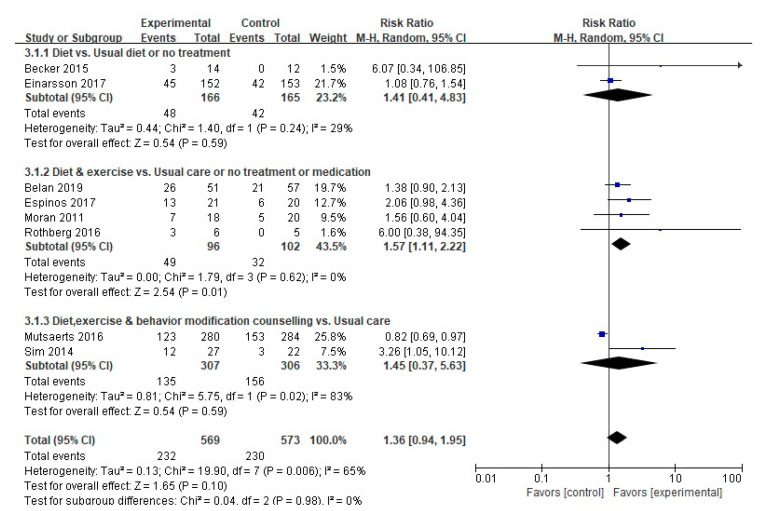
Forest plot of nonpharmacological interventions on the live birth rate. M-H, the Mantel-Haenszel method; Random, random effects model; CI, confidence interval.

A meta-analysis was performed of eight out of the 11 studies that measured the live birth rates, excluding those with a nonpharmacological intervention in the control group (*n* = 1), an intervention with a combined medication *(n* = 1), or no comparison between the experimental and the control groups (*n* = 1). No meaningful differences in the live birth rates were found between the experimental group and the control group (232/569 (40.8%) vs. 230/573 (40.1%); RR, 1.36; 95% CI, 0.94–1.95; *p* = 0.10). A subgroup analysis was performed due to medium heterogeneity (Higgins I^2^ = 65%) (Figure 6). The live birth rates were meaningfully higher in the experimental groups than in the control groups in the four studies that involved interventions combining diet and exercise (49/96 (51.04%) vs. 32/102 (31.4%); RR, 1.57; 95% CI, 1.11–2.22; *p* = 0.01), with no heterogeneity (Higgins I^2^ = 0%).

#### 3.4.4. Miscarriage Rate

**Figure 7:**
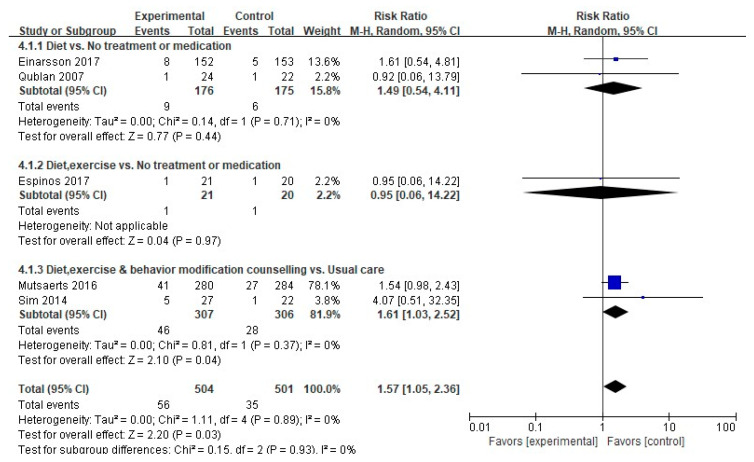
Forest plot of nonpharmacological interventions on the miscarriage rate. M-H, the Mantel-Haenszel method; Random, random effects model; CI, confidence interval.

A meta-analysis was performed on five of the nine studies that measured the miscarriage rate as a primary variable, excluding those with a nonpharmacological interventions in the control group (*n* = 1), an intervention with a combined medication (*n* = 1), no comparison between the experimental and the control groups (*n* = 1), or zero events *(n* = 1). A meaningfully higher miscarriage rate was found in the experimental group than in the control group (56/504 (11.1%) vs. 35/501 (7.0%); RR, 1.57; 95% CI, 1.05–2.36; *p* = 0.03), with no heterogeneity (Higgins I^2^ = 0%) (Figure 7). A subgroup analysis according to the type of intervention demonstrated a meaningful difference in the miscarriage rate in the two studies that involved diet, exercise, and behavioral modification counseling compared to the usual care (46/307 (44.0%) vs. 28/306 (50.8%); RR, 1.61; 95% CI, 1.03–2.26; *p* = 0.04), with no heterogeneity (Higgins I^2^ = 0%). 

### 3.5. Publication Bias

**Figure 8:**
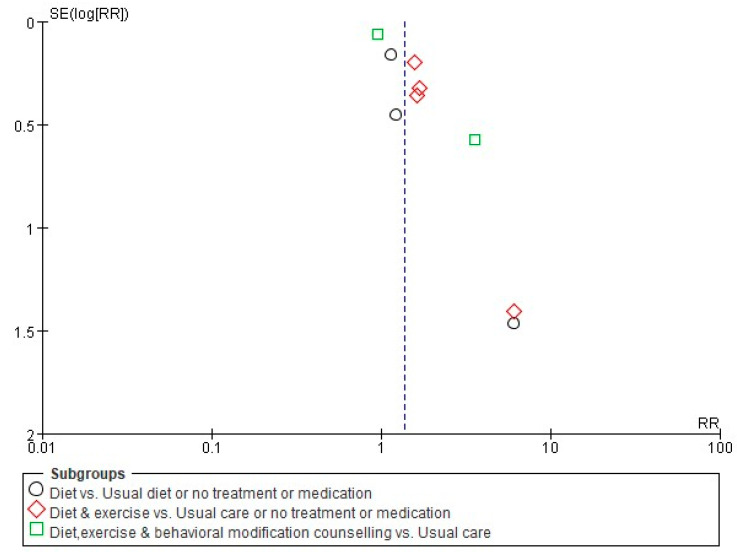
Funnel plots of the effects of nonpharmacological intervention on pregnancy rate.

The visual inspection of the funnel plot for asymmetry revealed a potential publication bias, which was confirmed by the Begg Mazumdar’s rank correlation test and Egger’s linear regression test. The results of the Egger test and Begg test showed a large discrepancy that the Egger test suggested was evidence of publication bias (Intercept 1.74, *t* = 5.41, *p*-value = 0.001) but not the Begg test (Kendall’s tau 0.14, *p* = 0.60) (Figure 8).

## 4. Discussion

This study investigated a broad range of nonpharmacological interventions, including diet, and obtained evidence of the effects of those interventions on primary reproductive outcomes in overweight and obese women with infertility. A more detailed discussion of the results is presented below.

### 4.1. Characteristics and Quality Assessment of Nonpharmacological Studies on Overweight and Obese Infertile Women 

An analysis of the year of publication of the 21 studies analyzed herein suggests that the number of RCTs on overweight and obese women affected by infertility has grown since the first study was reported in 2005. Previous studies mainly focused on interventions during pregnancy, but interest has shifted toward preconception nonpharmacological strategies as the first-line treatment for obese women due to the increased prevalence of infertility in obese women [45,46,47].

Dietitians, nurses/midwives, and physicians played the largest roles in the interventions. Most nonpharmacological interventions in infertile women with obesity were based on dietary counseling received from a dietitian. Additionally, they consulted with other specialists such as nurses or midwives and physicians in a variety of clinical settings. Health professionals’ positive perceptions of their health-promoting role for obese women can affect the successful implementation of an intervention [48,49]. Therefore, it is necessary to recognize the leading responsibility of these experts and to collaborate with various experts to successfully provide nonpharmacological interventions for overweight or obese infertile women.

The types of nonpharmacological interventions included dietary-only interventions; dietary and exercise-based interventions; a combination of diet, exercise, and behavioral modification; and lifestyle interventions combined with medication to achieve weight loss. This finding supports recent reports that dietary, exercise, or combined interventions are often used as nonsurgical interventions for overweight or obese women who experience infertility [50]. We identified two studies that provided supplementations (Chinese herbal medicine and omega-3 fatty acids). Accumulating evidence suggests that oral supplementations with two insulin-sensitizing agents, Myo-inositol (MI) and D-chiro-inositol (DCI) or their combination, can improve metabolic profiles and ovarian function in women with PCOS, although evidence is still lacking for pregnancy and birth outcomes [51,52,53,54]. However, in this study, we could not identify inositol-related studies; there was insufficient evidence to support the use of supplementations for overweight or obese women with subfertility. 

The results of these studies are difficult to evaluate, and more studies are needed to draw more definitive conclusions on these nonpharmacological interventions.

The nonpharmacological interventions mostly lasted for 13 weeks to six months, and the duration of follow-up was between seven and 12 months, which seems optimal considering that three to six months is an appropriate period to provide dietary and exercise interventions aimed at improving the initial weight loss and minimizing dropout rates [55].

The most commonly used outcome variables to measure the effects of nonpharmacological interventions were body weight, BMI, pregnancy rate, waist circumference, and the live birth rate, in-line with the primary outcome variables used to confirm the effects of pharmacological interventions [55,56,57,58]. This means that nonpharmacological interventions for overweight and obese infertile women focus on eliciting positive effects on actual weight loss and, ultimately, on pregnancy and birth, just like pharmacological interventions. Meanwhile, some studies included reported psychological and cognitive behavioral outcomes that are not generally touched upon by studies analyzing pharmacological interventions, which can be seen as evidence that nonpharmacological interventions are at the vanguard of efforts to address the high rates of depression, anxiety, stress, low quality of life, self-esteem, and body image issues experienced by overweight and obese infertile women [59]. Unfortunately, the psychological and cognitive effects of these interventions could not be collectively analyzed due to the different types of nonpharmacological interventions and limited number of studies that provided measurements of outcome variables. 

The methodological quality assessment of 14 out of the total of 21 studies and seven of the nine studies included in the meta-analysis showed a low risk of bias (for at least four of the seven domains), making them appropriate for a meta-analysis aimed at assessing a comprehensive range of outcomes. The risk of bias was the lowest in the selective reporting (reporting bias) domain, as most studies reported a clear protocol that was followed closely to report outcomes without omissions. The risk of bias was the highest in the domains related to blinding of the participants and researchers, which was most likely because blinding was inherently difficult for these interventions. 

### 4.2. Effects of Nonpharmacological Intervention Programs

The results of this study demonstrated that nonpharmacological interventions meaningfully increased the pregnancy rates. Interventions that combined diet and exercise were particularly effective in boosting pregnancy rates compared to the usual care or no treatment [50]. Meanwhile, a sensitivity analysis suggested that the study of Mutsaerts (2016), which provided diet, exercise, and behavioral modification counseling, contributed to increased heterogeneity in the effects of nonpharmacological interventions (Figure S1 in Supplementary File 3). This was probably because the experimental group in that study received a lifestyle intervention for six months, followed by 18 months of infertility treatment, while the control group received prompt infertility treatment without delay, and the effects were confirmed after a 24-month follow-up period. This difference in the timing of the infertility treatment likely had a major effect on the higher pregnancy rate found in the control group than in the experimental group. 

This study verified that nonpharmacological interventions meaningfully increased the natural conception rate. The number of studies on the combination of diet and exercise was too limited to confirm the comprehensive effects of those interventions, while dietary-only and diet, exercise, and behavioral modification counseling interventions improved the natural conception rates. A meaningful finding of this meta-analysis is that nonpharmacological interventions improved the natural conception rates, considering that previous studies did not report significant effects (Best et al., 2017), although some individual studies found a trend for increased natural conception in patients who receive weight loss interventions. These results support the guideline that nonpharmacological interventions should be used as the first-line treatment prior to infertility treatment for overweight and obese women experiencing infertility [60,61].

Nonpharmacological interventions were not proven to have a meaningful effect on the live birth rates, but the subgroup analysis suggested that interventions combining diet and exercise significantly increased the live birth rates compared to the usual care or no treatment. Previous studies also have not found that weight loss interventions increased the live birth rates [50]. A sensitivity analysis confirmed that the study of Mutsaerts (2016) was the main contributor to heterogeneity in the intervention effects, as seen from the pregnancy rates (Figure S2 in Supplementary File 3). Relatively few studies reported the live birth rates, which is presumably due to the high attrition rate in patients receiving nonpharmacological interventions and the difficulty of performing longitudinal studies to confirm the effects of nonpharmacological interventions on post-pregnancy outcomes and the birth rates. 

However, nonpharmacological interventions meaningfully increased the miscarriage rates, especially the diet, exercise, and behavioral modification counseling. The study of Mutsaerts (2016), which had a relatively high weighted effect, reported a relatively high baseline median BMI of the participants in the experimental group (>35 kg/m2) and a relatively low mean weight loss (4.4 ± 5.8 kg) at six months postintervention. As previous studies have suggested that more severe obesity leads to a higher risk of pregnancy loss [62], the severity of the participants’ obesity probably caused the higher risk of miscarriage. In a sensitivity analysis excluding the study of Mutsaerts (2016), the experimental group did not have a meaningfully higher risk of miscarriage than the control group (Figure S3 in Supplementary File 3). This is consistent with the results of previous studies suggesting that weight loss is unrelated to an increased risk of miscarriage [50,63]. Careful interpretation is needed through further studies to confirm these findings, given that only five RCTs were included in the meta-analysis of the miscarriage rate.

Potential evidence of publication bias was detected for the effects of nonpharmacological interventions by the funnel plot and Egger’s test. However, Cochrane suggests that a funnel plot, Begg test, and Egger’s linear regression test for analyzing publication bias be applied when more than 10 studies are analyzed in a meta-analysis, because due to the small number of studies included in the analysis, the power of the analysis could be low [64]. In addition, the factors of asymmetry could be due to a large heterogeneity between studies or large effects of an intervention other than publication bias.

The limitations of this study include possible publication bias for nonpharmacological intervention effects, although it is difficult to assess the publication bias due to the small number of studies included in the meta-analysis, the medium-to-high heterogeneity of the effect sizes for the pregnancy rates and live birth rates of all nonpharmacological interventions, requiring careful interpretation of the results, and the failure to sufficiently explore the psychological effects of nonpharmacological interventions due to the limited number of studies for each outcome variable. 

## 5. Conclusions

In conclusion, nonpharmacological interventions involving diet and exercise could have a positive effect on pregnancy rates and seem to be useful for improving live birth rates in overweight or obese infertile women. In addition, dietary interventions alone and interventions combining diet, exercise, and behavioral modification counseling could be beneficial for natural conception. 

Based on the outcomes of this study, more follow-up studies that include psychological indicators in addition to pregnancy-related markers as outcome variables should be carried out to establish the comprehensive effects of nonpharmacological interventions for overweight and obese women experiencing infertility.

## Figures and Tables

**Table 1:** Descriptive summary of included studies (*N* = 21).

Author (year)	Country (Study Design)	Inclusion Criteria	Population		Intervention						Follow-Up Period	Main Outcome Variables
			Intervention at Baseline	Control at Baseline	Description by Group (a~d)	Health Provider	Setting	Format (Individual/Group)	Duration	Number of Contacted		
**I. Diet-based intervention**												
**I-1. Diet vs. Others**												
Qublan et al. (2007) [24]	Jordan	Infertility Primary with PCOS CC-Resistant Age <36 BMI >29	*n* = 24 Age: 31.5 BMI: 32.2 PCOS	N=22 Age: 30.8 BMI: 31.9 PCOS	a: Diet (a 1200-1400 kcal/Day; 25% Proteins; 25% Fat; 50% Carbohydrates per Week) b: Met (850mg Twice Daily)	NR	Hospital (Single Center)	In Person	6 months	6+	12 months	A: BMI B: Pregnancy, Miscarriage Rate C: Ovulation (Rate), Menstrual Cycle D: FSH, LH, T, A, DHEAS, Estradiol E: Fasting Glucose, Fasting Insulin
Becker et al. (2015) [25]	Brazil	Infertility Planning IVF BMI 25-40	*n* = 14 Age:31.4 BMI:28.7 Multiple Factor	*n* = 12 Age: 31.3 BMI:28.8 Multiple factor	a: Diet (Hypocaloric Diet with Low-Glycemic Index/Low Glycemic Load Diet b: Usual Diet	Dietitian	Hospital (Single Center)	In Person (Individual)	12 weeks	3	24 months	A: BMI, BW, WC B: Pregnancy, Natural Conception, Live Birth Rate C: Oocytes Retrieved (No.) D: FSH, LH, T, SHBG, Estradiol E: Fasting Glucose, Fasting Insulin, HOMA-IR, Lipids G: Diet Intake
Einarsson et al. (2017) [26]	Nordic Countries	Infertility Planning IVF Age 18–38 BMI 30–34.9	*n* = 152 Age:31.5 BMI:33.1 Multiple Factor	*n* = 153 Age: 31.7 BMI:33.0 Multiple Factor	a: LCD Liquid Formula Diet (880kcal/Day for 12 Weeks, Weight Maintenance for 2–5 Weeks) before IVF b: Only IVF	Health Professional, Dietitian	9 Infertility Clinics	In Person (Individual)	16 weeks	6	NR	A: BMI, BW B: Pregnancy, Natural Conception, Live Birth, Miscarriage Rate C: Oocytes Retrieved (No.)
I-2. Diet (Type of diet)												
Turner-McGrievy et al. (2014) [27]	USA (Pilot Study)	Infertility Due to PCOS Trying to Conceive over 6 mo Age 18–35 BMI 25–49.9	*n* = 9 Age: 28.1 BMI: 42.7 PCOS	*n* = 9 Age: 27.4 BMI: 37.2 PCOS	a: Vegan Diet (a Low-Fat, Low–Glycemic Index Vegan Diet with no Caloric restriction) b: LCD ( 1200 kcal/Day, <90 kg; 1500 kcal/Day, >90kg)	Dietitian	Local Medical Clinic (Single Center)	In Person (Individual) + Email + Social Media	6 months	27+	6 months	A: BW B: Pregnancy Rate C: Ovulation (Detection), Menstrual Cycle F: QoL G: Diet Intake, Physical Activity
Galletly et al. (2007) [28]	Australia (Pilot Study)	Infertility Due to PCOS (Including Actively Try to Conceive) BW <140 kg	*n* = 12 Age: 33.0 BMI: 37.6 PCOS	*n* = 13 Age: 32.0 BMI:37.2 PCOS	Diet (6000KJ/Day for 12 Weeks, Weight Maintenance for 4 Weeks) + Exercise a: HPLC Diet (30% Protein, 40% Carbohydrate, 30% Fat) b: LPH Diet (15% Protein, 55% carbohydrate, 30% fat)	Dietitian	NR	In Person (Individual + Group)	16 weeks	16+	16 weeks	A: BMI, BW F: Depression and Anxiety G: Self-Esteem
I-3. Diet Plus Exercise Intervention												
Moran et al. (2011) [29]	Australia (Pilot Study)	Infertility Undergoing IVF (Previously had One Round of ART) Age 18–40 BMI 28–45	*n* = 18 Age: 33.8 BMI:34 Multiple Factor	*n* = 20 Age: 32.5 BMI:33.9 Multiple Factor	a: Diet (One Liquid Meal Replacement + 200 ml Reduced Fat Milk (1057 kJ)) + Exercise b: Standard Advice on Diet and Lifestyle Factors	Dietitian	Hospital (Single Center)	In person (Individual) + Phone	52.6 (14.0)/53.5 (16.6) Days	3	NR	A: BMI, BW, WC B: Pregnancy, Live Birth Rate
Rothberg et al. (2016) [30]	USA (Pilot Study)	Infertility due to Ovulation Dysfunction Age 18-40 BMI 35-45	*n* = 6 Age: 33 BMI:41 Multiple Factor	*n* = 5 Age: 30 BMI:41 Multiple Factor	a: VLED (Liquid Meal Replacements, 800 Kcal/Day for 12 Weeks) + Low-Calorie Conventional Food-Based Diet (CFD) (for 4 Weeks) + Exercise Encouragement b: CFD (for 16 Weeks)	Dietitian, Physician	Academic Institution (Single Center)	In Person	16 weeks	12	12 months	A: BMI, BW, WC B: Pregnancy, Live Birth, Miscarriage Rate C: Ovulation (Induction, Detection), E: Fasting Glucose, Fasting Insulin, HOMA-IR, Lipids F: QoL, Depression
Belan et al. (2019) [31]	Canada	Infertility Age 18–40 Obesity with BMI ≥30 Overweight if PCOS, BMI ≥27	*n* = 51 Multiple Factor (PCOS, Non-PCOS)	*n* = 57 Multiple Factor (PCOS, Non-PCOS)	a: Interdisciplinary Lifestyle Intervention (Diet + Exercise) before Infertility Treatment b: Standard Fertility Treatments	Dietitian, Kinesiologist, Psychologists	Academic Hospital (Single Center)	In person (Group + Individual) + Phone/Email	18 months or Until the End of Pregnancy	25	18 months or Until the End of Pregnancy	A: BW, WC B: Pregnancy, Natural Conception, live birth rate G: Diet Intake, Physical Activity
Espinos et al. (2017) [32]	Spain (Pilot Study)	Infertility Presenting First IVF Age 18–37 BMI 30-40	*n* = 21 Age:32.0 BMI:34.6 Multiple Factor	*n* = 20 Age:32.9 BMI:34.0 Multiple Factor	a: Diet (Reduce Total Daily Calorie Intake by at least 500–800 kcal) + Exercise before Single IVF or ICSI b: No Intervention	Dietitian, Trained Staff	Hospital	In Person (Individual)	12 weeks	36+	NR	A: BMI, BW, WC B: Pregnancy, Live Birth, Miscarriage Rate C: Oocytes Retrieved (No.)
II. Diet, Exercise plus behavioral modification intervention												
Mutsaerts et al. (2016) [33]	Netherlands	Infertility Age 18–39 BMI >29	*n* = 280 Age:29.7 BMI: † 36.0 Multiple Factor	*n* = 284 Age:29.8 BMI: † 36.0 Multiple Factor	a: Diet, Exercise, Behavioral Change (Motivational Counselling) before Infertility Treatment b: Conventional Infertility Treatment (with Dutch Infertility Guideline)	Nurse	6 University Medical Centers and 17 General Hospitals	In Person (Individual)+Phone/Email	6 months	10	24 months	A: BW, WC B: Pregnancy, natural conception, live birth, miscarriage rate, C: Ovulation (induction) H: Adverse outcome
Oberg, et al. (2019) [34]	Sweden (Pilot Study, Pre-Post design)	Infertility due to PCOS Aged 18–40 BMI ≥27	*n* = 34 Age: 31.0 BMI:33.5 PCOS	*n* = 34 Age: 29.9 BMI:34.3 PCOS	a: Behavioral Modification (Information of Weight Control, Personal Leadership, Mindfulness and Physical Activity) b: Minimal Intervention (General Healthy Lifestyle Recommendations)	Lifestyle Coach (PhD in Endocrinology, Metabolism) and Midwife	Hospital	In Person (Small Groups + Individual)	16 weeks	16	12 months	A: BMI, BW B: Pregnancy, Natural Conception, live Birth, Miscarriage Rate C: Ovulation (Rate), Menstrual Cycle D: FSH, LH, T, A, SHBG, FAI, DHEAS, Estradiol E: Fasting Glucose, Fasting Insulin, HOMA-IR
Jiskoot et al. (2019) [35]	Netherlands	Infertility due to PCOS Trying to Conceive over 1 year Age 18–38 BMI > 25	a: *n* = 72 PCOS	b: *n* = 77 c: *n* = 60 PCOS	a: CBT Lifestyle Intervention (Diet + Exercise) b: CBT +Short Message Service (SMS) c: (Control) Usual Care (Encouraged to Lose Weight by Publicly Available Services)	Mental Health Professional, Physical Therapist and Dietitian	Academic Hospital	In Person (Group + Individual) + Phone	12 months	25	12 months	A: BMI, BW F: Depression G: Self-Esteem, Body Image
Sant’Anna et al. (2017) [36]	NR	Infertility Age 18–48 BMI > 25	*n* = 51 Age: † 37.0	*n* = 49 Age: † 31.0	a: Mindfulness based Stress Reduction Intervention b: No Intervention All Participants Received Dietary Plan and Physical Exercise	Trained Personnel	Hospital	NR	8 weeks	8	12 weeks	A: BW, WC B: QoL
Sim et al. (2014) [37]	Australia (Evaluator-Blinded)	Infertility Intending to Commence IVF, ICSI or Cryo-Stored Embryo Transfer Age18–37 BMI ≥ 30	*n* = 27 Age:32.9 BMI:35.1 Multiple Factor	*n* = 22 Age:32.8 BMI:38.0 Multiple Factor	a: Diet (VLED for 6 Weeks then Hypocaloric Diet for 6 Weeks) Exercise and Psychological and Behavioral Advice then ART b: Standard Care (Advised to See their GP for Weight Loss) then ART	Fertility Fellow, Midwife, Fertility Counsellor, Dietitian	Hospital (Single Center)	In Person (Group + Individual)	12 weeks	13	12 months	A: BMI, BW, WC B: Pregnancy, Natural Conception, Live Birth, Miscarriage Rate H: Adverse Outcome
III. Lifestyle intervention combined medication												
Zhang, and Li (2017) [38]	China	Infertility due to PCOS Age 22–34	*n* = 51 Age: 28.3 BMI: 23.0 PCOS	*n* = 50 Age: 27.8 BMI:23.4 PCOS	a: Lifestyle Intervention (Low-Fat Diet + Exercise Strengthening) with Met (500 mg, Three Times Daily, 3 Days of Menstruation) + CC (50–100 mg Once Daily, 1st–3rd Days of 3–5 days of Menstruation) b: Met + CC Same Above	NR	Hospital (Single Center)	In Person	6 months	NR	6 months	A: BMI, BW B: Pregnancy Rate C: Ovulation (Rate), Menstrual Cycle D: LH, T E: Fasting Insulin, Lipids (TG)
Legro et al. (2015) [39]	USA	Infertility due to PCOS Planning Pregnancy) Age 18–40 BMI 27–42	a: *n* = 44 Age: a:28.6 BMI: a:35.1 PCOS	b: *n* = 43 c: *n* = 45 Age: b:28.7 c:29.8 BMI: b:35.5 c:35.1 PCOS	a: Lifestyle Intervention (Meal Replacements+ Exercise to Achieve >7% Weight Loss) + Weight Loss Medication (Sibutramine or Orlistat) before Ovulation Induction b: (Combined) a +c c: (Control) OCPs Only before Ovulation Induction	Trained Study Coordinators	Academic Health Centers (Two-Site)	In Person	16 weeks	NR	13 months	A: BW, WC B: Pregnancy Rate, Live Birth Rate, Miscarriage Rate C: Ovulation (Rate) D: T, SHBG E: Glucose (2 h, AUC), Insulin (2 h, AUC), Insulin Sensitivity Index F: QoL H: Adverse Outcome
Palomba et al. (2010) [40]	Italy (Assessor-Blinded)	Infertility due to PCOS with Anovulatory Infertility and Known CC Resistance Overweight (BMI 25–30), Obese (BMI > 30)	a: *n*=32 Age: a:27.5 BMI: a:31.3 PCOS	b: *n* = 32 c: *n* = 32 Age: b:28.4 c:26.5 BMI: b:31.1 c:32.3 PCOS	a: Lifestyle Intervention (Structured Exercise Training (SET) + Hypocaloric Diet) b: (Combined) Lifestyle Intervention + CC (One Cycle, for First 2 Weeks) c: (control) CC	Cardiologist	NR (Single Center)	In Person	6weeks	21+	6 weeks	A: BMI, BW, WC B: Pregnancy Rate C: Ovulation (rate) D: FSH, LH, T, SHBG, FAI, A, DHEAS, Estrogen E: Fasting Glucose, Fasting Insulin, HOMA-IR
Karimzadeh and Javedani (2010) [41]	Iran (Double-Blind)	Infertility Primary with PCOS Age 19–35 BMI 25–29.9	a: *n* = 90 b: *n* = 90 Age: a:27.5 b:27.3 BMI: a:27.2 b:27.2 PCOS	c: *n* = 88 d: *n* = 75 Age: c:27.3 d:27.5 BMI:c:28.0 d:.27.9 PCOS	a: CC (100 mg on Days 3-7) b: Met (Initial dose of 500 mg Increased Until 1500 mg/day for 3–6 Months) c: CC + Met d: Lifestyle Modification (Low Calorie Diet + Exercise Advice) only	Dietician	University-Based infertility Clinic and Research Center	In Person	NR	2+	8 months	A: BMI, WC B: Pregnancy Rate C: Menstrual Cycle D: T, SHBG E: Insulin, Lipids
Tang et al. (2006) [42]	UK (Double-Blind)	Infertility due to Anovulatory PCOS Including Desire to Conceive Age 18–39 BMI > 30	*n* = 56 Age: 29.7 BMI:37.6 PCOS	*n* = 66 Age: 29.8 BMI:38.9 PCOS	a: Lifestyle Intervention (Hypocaloric Diet; Reduction in Daily Intake by 500 kcal, + Exercise Advice) + Met 850 mg Twice Daily b: Placebo + Lifestyle Intervention	Dietitian, Nurse and Medical Personnel	8 Hospital Infertility Clinics	In Person (Individual)	6 months	6+	6 months	A: BMI, BW, WC B: Pregnancy Rate C: Menstrual Cycle D: T, SHBG, FAI E: Fasting Glucose, Fasting Insulin, QUICKI, Lipids
IV. Supplementation												
Arentz et al. (2017) [43]	Australia	Infertility due to PCOS) Including Desire to Conceive Age 18–44 BMI ≥ 24	*n* = 60 Age: 29.2 BMI:34.1 PCOS	*n* = 62 Age: 28.9 BMI:35.2 PCOS	a: Herbal Medicine + Lifestyle Intervention (Dietary + Exercise Behaviors) b: Lifestyle Intervention	Dietitian, Exercise Physiologist, Herbal Practitioner	Community	In Person (Individual)	12 weeks	4+	12 weeks	A: BMI, BW, WC B: Pregnancy, Live Birth, Miscarriage Rate C: Menstrual Cycle D: FSH, LH, T, SHBG, FAI E: Fasting Glucose, Fasting Insulin, Insulin Sensitivity (QUICKI) F: QoL, Depression, Anxiety, and Stress
Nadjarzadeh et al. (2015) [44]	Iran (Double Blind)	Referred to Infertility Center Due to PCOS Age 20–40 BMI 25–40	*n* = 39 Age: 26.9 BMI:31.5 PCOS	*n* = 39 Age: 26.9 BMI: 31.9 PCOS	a: Omega-3 (180 mg EPA and 120 mg DHA), 3 Capsules Daily) b: Placebo (1 g Paraffin, 3 Capsules Daily)	NR	Hospital	In Person (Individual) + Phone	8 weeks	10	8 weeks	A: BMI D: FSH, LH

Note: † Median, age: mean age (year), BMI = body mass index (kg/m^2^), BW = body weight (kg), WC = waist circumference (cm), FSH = follicle stimulating hormone, LH = luteinizing hormone, T = testosterone, A = androstenedione, DHEAS = dehydroepiandrosterone sulfate, HOMA-IR = homeostatic model of assessment of insulin resistance, SHBG = sex hormone-binding globulin, FAI = free androgen index, QUICKI = insulin sensitivity check index method, IVF = in vitro fertilization, ICSI = intracytoplasmic sperm injection, ART = assisted reproductive technology, PCOS = polycystic ovary symptom, CC = clomiphene citrate, Met = metformin, LCD = low-calorie diet, HPLC = high-protein, low-carbonate, LPHC = low-protein, high-carbonate, VLED = very low energy diet, OCPs = oral contraceptive pills, CBT = cognitive behavioral therapy, No. = number, NR = not reported, QoL = health-related quality of life, A = anthropometric, B = pregnancy and birth outcome, C = fertility-related outcome, D = reproductive hormone, E = metabolic hormone, F = psychological outcome, G = cognitive behavioral outcome, and H = adverse outcome.

**Table 2:** Characteristics of the included studies *(n* = 21).

Characteristics	Categories	*n* (%)
Year	2005–2009	3 (14.3)
	2010–2014	5 (23.8)
	2015–2019	13 (61.9)
Country	Australia USA Netherland Nordic Countries (Sweden, Denmark, and Iceland) Iran Canada UK Italy Spain China Brazil Jordan Unclear	4 (19.0) 3 (14.3) 2 (9.5) 2 (9.5) 2 (9.5) 1 (4.8) 1 (4.8) 1 (4.8) 1 (4.8) 1 (4.8) 1 (4.8) 1 (4.8) 1 (4.8)
Sample Size of Each Group	5–29 30–49 50–99 100–149 150–200 200–284	8 (38.1) 4 (19.0) 6 (28.6) 1 (4.8) 1 (4.8) 1 (4.8)
Mean Age of Participants in Each Group (Year)	25–29.9 30–37 Not reported	9 (42.9) 10 (47.6) 2 (9.5)
Mean Body Mass Index of Participants in Each Group (kg/m^2^)	23.0-29.9 30.0–34.9 35.0–42.7 Not reported	3 (14.3) 8 (38.1) 7 (33.3) 3 (14.3)
Infertility Factor of Participants	Polycystic ovary symptom only Multiple Not reported	12 (57.1) 8 (38.1) 1 (4.8)
Type of Intervention	Diet-based intervention Diet vs. Others Diet (Type of Diet) Diet plus exercise intervention Diet, exercise plus behavioral modification Lifestyle intervention combined medication Supplementation	5 (23.8) 3 (14.3) 2 (9.5) 4 (19.0) 5 (23.8) 5 (23.8) 2 (9.5)
Health Provider Involvement † (*n* = 35)	Dietitian Physical professional (Physiologist or Kinesiologist or Physical Therapist) Nurse or Midwife Physician Metal health Professional Trained Personnel Not specified Health Professional Herbal Practitioner Counsellor Not Reported	13 (37.1) 3 (8.6) 4 (11.4) 4 (11.4) 2 (5.7) 2 (5.7) 2 (5.7) 1 (2.9) 1 (2.9) 3 (8.6)
Setting	Hospitals or Infertility Clinics Academic Institution Community Not Reported	16 (76.2) 2 (9.5) 1 (4.8) 2 (9.5)
Format † *(n* = 29)	In Person Individual Mixed (Individual + Group) Unclear Phone Email Social Media (Facebook) Not Reported	20 (69.0) 9 (31.0) 5 (17.2) 6 (20.7) 4 (13.8) 3 (10.3) 1 (3.4) 1 (3.4)
Duration of Intervention (Weeks or Months)	7.5 Weeks–12 Weeks 13 Weeks–6 Months 7–12 Months 13–18 Months Not Reported	8 (38.1) 10 (47.6) 1 (4.8) 1 (4.8) 1 (4.8)
Follow-up period (Weeks or Months)	6 Weeks–12 Weeks 13 Weeks–6 Months 7–12 Months 13–24 Months Not Reported	4 (19.0) 4 (19.0) 6(28.6) 4 (19.0) 3(14.3)

† Duplicated answers.

**Table 3:** Characteristics of the outcome variables.

Categories	Outcome Variables (Measurements)	*n*
Anthropometrics (*n* = 56)	Body weight	18
	BMI	16
	Waist Circumference	13
	Hip Circumference	2
	WHR	6
	Ferriman-Gallwey score	1
Pregnancy and Birth outcomes (*n* = 62)	Pregnancy rate	17
	Natural conception	6
	Infertility treatments conception (IVF, ICSI, IUI, ovulation induction etc.)	4
	Multiple pregnancy	7
	Ectopic pregnancy	3
	Miscarriage	9
	Live birth	11
	Gestational age at delivery	2
	Infant birth weight	2
	Delivery mode	1
Fertility-related outcomes (*n* = 45)	Ovulation- Ovulation rate- Ovulation detection	52
	- Regularity of menstrual cycles	7
	Fertility treatment measures	
	- Number of oocytes retrieved	3
	- Number of medication-induced cycles of ovulation	2
	- Total dose of FSH used	2
	- Number of assisted conception cycles	2
	- Fertilization rate	2
	- Implantation rate1	2
	- No. of good quality embryos	2
	- Fresh transfer (IVF or ICSI)	3
	- Cryo-stored embryo transfer	3
	- Cancelled cycle	2
	Ultrasound parameters	
	- Antral follicle count	2
	- Ovarian volume	3
	- Endometrial thickness	3
Reproductive Hormone outcomes (*n* = 58)	Gonadotropin Hormone- FSH- LH - LH:FSH ratio	673
	Prolactin	4
	Androgen Hormone - Testosterone - Androstenedione - DHT - DHEAS - Free Androgen Index	9 3 1 3 4
	Hirsutism	1
	SHBG	7
	Anti-Mullerian hormone	2
	Estrogens - Estrogen - Estradiol	1 3
	Progesterone	2
	17-OHP	2
Metabolic outcomes (*n* = 57)	Glucose - Fasting glucose - 2 h glucose - AUC glucose	7 1 1
	Insulin - Fasting Insulin - 2 h insulin - AUC insulin - GIR (fasting glucose-to-insulin ratio) - Insulin sensitivity (QUICKI) - Insulin sensitivity Index - Insulin resistance (HOMA-IR)	9 1 1 1 2 1 4
	Lipid profile - Total cholesterol - Triglycerides - High-density lipoprotein (HDL) - Low-density lipoprotein (LDL) - Visfatin - Adiponectin	3 4 2 3 1 1
	Thyroid Hormone - TSH - Free T4	1 1
	Others - Acylated ghrelin - Leptin - Hs-CRP	1 1 1
	Fat mass	4
	Blood pressure	5
	Heart rate	1
Psychological outcomes (*n* = 9)	Quality of life - (PCOSQ) - (EQ-5D) - (PGWBI)	3 1 1
	Depression - (BDI-II) - (IDS-SR)	1 1
	Depression and anxiety (HAD) Depression, anxiety, and stress (DASS21)	1 1
Cognitive Behavioral outcomes (*n* = 9)	Self-esteem (SE)	2
	Body image (FNAES)	1
	Physical activity - (PPAQ) - Accelerometer	1 2
	Dietary intake - (health eating index) - (ASA24) - Calculated by Nutribase7software (CyberSoft Incorporated, Phoenix, Arizona, USA)	1 1 1
Adverse outcomes (*n* = 11)	During preconception (diarrhea, steatorrhea, breast pain, abdominal pain, dysmenorrhea, abnormal and uterine bleeding)	2
	During infertility treatment (headache, OHSS, torsion, bleeding infection, etc.)	2
	During pregnancy (GDM, gestational HTN, pre-eclampsia, and Cesarean section)	4
	During postpartum Adverse postpartum outcome (PPH and total perineal rupture) Adverse neonatal outcome (congenital anomalies, premature birth, SGA, LGA, Apgar score <7 at 5 min, death, stillbirth, etc.)	2 1

Note: BMI = body mass index, WHR = waist–hip ratio, IVF = in vitro fertilization, ICSI = intracytoplasmic sperm injection, IUI = intrauterine insemination, FSH = follicle-stimulating hormone, LH = luteinizing hormone, DHT = dihydrotestosterone, DHEAS = dehydroepiandrosterone sulfate, HOMA-IR = homeostatic model of assessment of insulin resistance, SHBG = sex hormone-binding globulin, FAI = free androgen index, QUICKI = insulin sensitivity check index method, 17-OHP = 17-hydroxyprogesterone, AUC = area under the concentration-time curve, TSH = thyroid-stimulating hormone, Free T4 = free thyroxine, Hs-CRP = high-sensitivity C-reactive protein, PCOSQ = quality of life questionnaire measures for polycystic ovary syndrome, EQ-5D = EuroQoL-5 dimensions, PGWBI = psychological general well-being index, BDI-II = Beck depression inventory II, IDS-SR = the inventory of depressive symptomatology self-report, HAD = the hospital anxiety and depression rating scale, DASS21 = the depression, anxiety, and stress scale, SE = the Rosenberg self-esteem rating scale, FNAES = fear of negative appearance evaluation scale, PPAQ = Paffenbarger physical activity questionnaire, ASA24 = Automated Self-administered 24-h recall, OHSS = ovarian hyperstimulation syndrome, GDM = gestational diabetes mellitus, HTN = hypertension, PPH = postpartum hemorrhage, SGA = small for gestational age, and LGA = large for gestational age.

## Supplementary Materials

The following are available online at https://www.mdpi.com/1660-4601/17/20/7438/s1, Table S1: Search Strategy on Ovid MEDLINE: Table S2: Excluded studies; Figure S1: Post hoc sensitivity analysis-pregnancy rate, Figure S2: Post hoc sensitivity analysis-live birth rate, Figure S3: Post hoc sensitivity analysis-miscarriage rate.
