# Supplementary material for: Effectiveness of Non-Pharmacological Interventions for Overweight or Obese Infertile Women: A Systematic Review and Meta-Analysis
Source: Int J Environ Res Public Health. 2020 Oct 13;17(20):7438. doi: 10.3390/ijerph17207438 (PMC7650570; doi:10.3390/ijerph17207438)
Supplement: Supplementary file 1 [file ijerph-17-07438-s001.zip › supplementary file_S1.pdf]

International Journal of
Environmental Research and Public Health

MDPI

Supplementary File S1: Search Strategy on Ovid MEDLINE.

Table S1. Search strategy in Ovid MEDLINE.

SET	Ovid MEDLINE (Results)
1	exp Embryo Transfer/ (15545)
2	exp Reproductive Techniques/ (145200)
3	exp Fertilization in Vitro/ (34533)
4	exp Infertility/ (63911)
5	exp Insemination, Artificial/ (11471)
6	exp Ovulation Induction/ (12756)
7	exp Sperm Injections, Intracytoplasmic/ (6320)
8	anovulation$.ab,ti. (2650)
9	artificial insemination$.ab,ti. (6452)
10	assisted reproduct$.ab,ti. (13979)
11	(asthenozoospermia or oligospermia or azoospermia).ab,ti. (7260)
12	childless$.ab,ti. (1777)
13	COH.ab,ti. (1651)
14	(desire adj3 child$).ab,ti. (1294)
15	Embryo Transfer$.ab,ti. (11368)
16	fecund$.ab,ti. (12731)
17	fertil$.ab,ti. (161212)
18	ICSI.ab,ti. (7935)
19	in vitro fertili?ation.ab,ti. (22128)
20	infecundit$.ab,ti. (53)
21	infertil$.ab,ti. (57571)
22	intracytoplasmic sperm injection$.ab,ti. (6846)
23	IUI.ab,ti. (1679)
24	IVF.ab,ti. (22504)
25	IVF-ET.ab,ti. (2240)

SET	Ovid MEDLINE (Results)
26	(ovari$ adj2 stimulat$).ab,ti. (6738)
27	(ovari$ adj2 induction).ab,ti. (283)
28	intrauterine insemination$.ab,ti. (2409)
29	ovulation$.ab,ti. (29477)
30	reproductive procedure.ab,ti. (17)
31	reproduction techniques.ab,ti. (796)
32	PCOS.ab,ti. (10336)
33	(polycyst$ adj2 ovar$).ab,ti. (15671)
34	sexual sterilit$.ab,ti. (17)
35	subfecund$.ab,ti. (143)
36	subfertil$.ab,ti. (4854)
37	superovulat$.ab,ti. (3338)
38	1 or 2 or 3 or 4 or 5 or 6 or 7 or 8 or 9 or 10 or 11 or 12 or 13 or 14 or 15 or 16 or 17 or 18 or 19 or 20 or 21 or 22 or 23 or 24 or 25 or 26 or 27 or 28 or 29 or 30 or 31 or 32 or 33 or 34 or 35 or 36 or 37 (362548)
39	exp Overweight/ (205669)
40	exp Thinness/ (5692)
41	exp Body Weight/ (443304)
42	exp "Body Weights and Measures"/ (581136)
43	exp Body Mass Index/ (119134)
44	(obesit$ or obese$ or overweigh$or over weigh$ or adiposit$or corpulen$ or body mass index or BMI or waist hip ratio$).ab,ti. (421443)
45	(body adj2 (weight$ or size$ or fat$)).ab,ti. (254733)
46	(waist circumference or skinfold thickness or thinness$ or underweigh$ or leanness$ or weight insufficien$).ab,ti. (40898)
47	(weight adj2 (reduc$ or los$ or control$ or manage$ or gain$)).ab,ti. (172215)
48	(Low weigh$ or Weight Loss or Low body weight$ or low-body-weight or Light-weight).ab,ti. (86745)
49	39 or 40 or 41 or 42 or 43 or 44 or 45 or 46 or 47 or 48 (1010668)
50	exp Body Weight Changes/ (67581)
51	exp Weight Reduction Programs/ (1882)

SET	Ovid MEDLINE (Results)
52	exp Nutrition Therapy/ (97183)
53	exp Diet Therapy/ (52099)
54	exp Health Education/ (234722)
55	exp Health Behavior/ (300443)
56	exp Psychotherapy/ (188560)
57	exp Behavior Therapy/ (71005)
58	exp Cognitive Behavioral Therapy/ (26232)
59	exp Counseling/ (42124)
60	exp Complementary Therapies/ (219329)
61	exp Exercise/ (181688)
62	exp Exercise Therapy/ (47018)
63	exp Life Style/ (87692)
64	exp Social Support/ (67748)
65	exp Nursing Care/ (132350)
66	(weight adj2 (management$ or intervention$ or program$ or education$ or counsel$ or advi$ or diet*)).ab,ti. (18352)
67	(diet$ adj2 (calori$ or energ$ or exercise$ or modif$ or management$ or intervention$ or therp$ or program$ or education$ or counsel$ or advi$)).ab,ti. (44972)
68	(nutrition$ adj2 (intervention$ or therp$ or program$ or education$ or counsel$ or advi$)).ab,ti. (18512)
69	(exercise$ or physical activit$ or physical fitnes$).ab,ti. (356480)
70	(lifestyle$ adj2 (modif$ or change$ or management$ or intervention$ or therp$ or program$ or education$ or counsel$ or advi$)).ab,ti. (24657)
71	(behavio$ adj2 (modif$ or change$ or management$ or intervention$ or therp$ or program$ or education$ or counsel$ or advi$)).ab,ti. (71157)
72	(psycho$ adj2 (therp$ or education$ or intervention$ or program$)).ab,ti. (21711)
73	(cogniti$ adj2 (therp$ or education$ or intervention$ or program$)).ab,ti. (6530)
74	(cognitive behavio$ adj2 (education$ or intervention$ or program$)).ab,ti. (2958)
75	cognitive behavioral therap$.ab,ti. (8324)
76	CBT.ab,ti. (9679)
77	social support$.ab,ti. (35833)

SET	Ovid MEDLINE (Results)
78	directive counsel$.ab,ti. (165)
79	(sex$ adj3 (counsel$ or therap$)).ab,ti. (4486)
80	prescriptive counsel$.ab,ti. (3)
81	motivational interview$.ab,ti. (3735)
82	stress management$.ab,ti. (4354)
83	(complementar$ adj2 (therap$ or treatment$ or techni$ or medicine)).ab,ti. (11842)
84	(alternat$ adj2 (therap$ or treatment$ or techni$)).ab,ti. (54858)
85	(mind body intervention$ or mind body program$).ab,ti. (332)
86	mindfulness.ab,ti. (6106)
87	aroma therap$.ab,ti. (50)
88	relaxation therap$.ab,ti. (634)
89	acupuncture$.ab,ti. (20641)
90	acupressure.ab,ti. (1012)
91	meditation$.ab,ti. (4600)
92	yoga$.ab,ti. (4234)
93	hypno$.ab,ti. (21507)
94	non?surgical$.ab,ti. (15321)
95	non?pharmacological$.ab,ti. (3735)
96	(nursing care$ or nursing intervention$ or Nursing Care Management$).ab,ti. (29869)
97	50 or 51 or 52 or 53 or 54 or 55 or 56 or 57 or 58 or 59 or 60 or 61 or 62 or 63 or 64 or 65 or 66 or 67 or 68 or 69 or 70 or 71 or 72 or 73 or 74 or 75 or 76 or 77 or 78 or 79 or 80 or 81 or 82 or 83 or 84 or 85 or 86 or 87 or 88 or 89 or 90 or 91 or 92 or 93 or 94 or 95 or 96 (1809631)
98	38 and 49 and 97 (3505)
99	exp Random Allocation/ (100017)
100	exp Controlled Clinical Trial/ (576359)
101	exp Randomized Controlled Trial/ (487993)
102	exp Clinical Trial/ (833435)
103	(randomised* or randomized*).ab,ti. (581281)
104	clinical trial.ab,ti. (129527)

SET	Ovid MEDLINE (Results)
105	(random* adj25 (trial* or stud* or investigat* or cross over or crossover)).ab,ti. (658827)
106	Controlled Clinical Trial.ab,ti. (13715)
107	Random Allocation.ab,ti. (1553)
108	99 or 100 or 101 or 102 or 103 or 104 or 105 or 106 or 107 (1337746)
109	98 and 108 (620)
110	limit 109 to humans (386)

© 2020 by the authors. Submitted for possible open access publication under the terms and conditions of the Creative Commons Attribution (CC BY) license (http://creativecommons.org/licenses/by/4.0/).
