# Supplementary material for: Effectiveness of Non-Pharmacological Interventions for Overweight or Obese Infertile Women: A Systematic Review and Meta-Analysis
Source: Int J Environ Res Public Health. 2020 Oct 13;17(20):7438. doi: 10.3390/ijerph17207438 (PMC7650570; doi:10.3390/ijerph17207438)
Supplement: Supplementary file 1 [file ijerph-17-07438-s001.zip › Supplementary File_S2.pdf]

Supplementary File S2: The reasons for exclusion of all studies that were excluded after a full-text review are listed below.

Table S2. Excluded studies (*N*=147).

	Study	Category for exclusion	Detail Reason(s) for Exclusion
1	Kadoura S.A. & Nattouf M. 2019 [S1]	Not relevant population	Ineligible for infertility criteria or not trying to conceive (PCOS), ineligible for obesity or overweight criteria
2	Jamilian M.Z. et al. 2018 [S2]	Not relevant population	Ineligible for infertility criteria or not trying to conceive (PCOS), ineligible for obesity or overweight criteria
3	Vizza L.S. et al. 2016 [S3]	Not relevant population	Ineligible for infertility criteria or not trying to conceive, ineligible for obesity or overweight criteria
4	Almenning I. et al. 2015 [S4]	Not relevant population	Ineligible for infertility criteria or not trying to conceive, ineligible for obesity or overweight criteria
5	Glueck C.J. et al. 2013 [S5]	Not relevant population	Ineligible for infertility criteria or not trying to conceive, ineligible for obesity or overweight criteria
6	Johansson J. et al. 2013 [S6]	Not relevant population	Ineligible for infertility criteria or not trying to conceive, ineligible for obesity or overweight criteria
7	Jedel E. L. et al. 2011 [S7]	Not relevant population	Ineligible for infertility criteria or not trying to conceive (PCOS), ineligible for obesity or overweight criteria
8	Wehr E.P. and Obermayer-Pietsch T. R. 2011 [S8]	Not relevant population	Ineligible for infertility criteria or not trying to conceive, ineligible for obesity or overweight criteria, non-RCT
9	Moran L.J. et al. 2006 [S9]	Not relevant population	Ineligible for infertility criteria or not trying to conceive (PCOS), ineligible for obesity or overweight criteria
10	Esmael M. & Nashed G.A. 2019 [S10]	Not relevant population	Ineligible for infertility criteria or not trying to conceive (PCOS)
11	Donne M., Alibrandi D., Papa A. & Benvenga M. 2019 [S11]	Not relevant population	Ineligible for infertility criteria or not trying to conceive (PCOS)
12	Tiwari N., Pasrija S. and Jain S. 2019 [S12]	Not relevant population	Ineligible for infertility criteria or not trying to conceive (PCOS)
13	Hiam D. et al. 2019 [S13]	Not relevant population	Ineligible for infertility criteria or not trying to conceive (PCOS)
14	Munir S. S. et al. 2018 [S14]	Not relevant population	Ineligible for infertility criteria or not trying to conceive (PCOS)

	Study	Category for exclusion	Detail Reason(s) for Exclusion
15	Abd Al Samea G.A., Mahmoud N. F. Hamada H.A. Gabr A.A.2018 [S15]	Not relevant population	Ineligible for infertility criteria or not trying to conceive (PCOS)
16	Miranda-Furtado C.L. et al. 2018 [S16]	Not relevant population	Ineligible for infertility criteria or not trying to conceive (PCOS)
17	Mani H. et al. 2018 [S17]	Not relevant population	Ineligible for infertility criteria or not trying to conceive (PCOS),
18	Lopes I. P. et al. 2018 [S18]	Not relevant population	Ineligible for infertility criteria or not trying to conceive (PCOS), secondary analysis of RCT
19	Lara L. et al. 2018 [S19]	Not relevant population	Ineligible for infertility criteria or not trying to conceive (PCOS)
20	Khan A. A. and Begum W. 2018 [S20]	Not relevant population	Ineligible for infertility criteria or not trying to conceive (PCOS)
21	Foroozanfard F. R. et al. 2017 [S21]	Not relevant population	Ineligible for infertility criteria or not trying to conceive (PCOS)
22	Matsuzaki T. D. et al. 2017 [S22]	Not relevant population	Ineligible for infertility criteria or not trying to conceive
23	Bauer J. L. et al. 2017 [S23]	Not relevant population	Ineligible for infertility criteria or not trying to conceive
24	McBreairty L.E. et al. 2017 [S24]	Not relevant population	Ineligible for infertility criteria or not trying to conceive (PCOS)
25	Lai L. et al. 2017 [S25]	Not relevant population	Ineligible for infertility criteria or not trying to conceive (PCOS)
26	Jafari-Sfidvajani S. et al. 2017 [S26]	Not relevant population	Ineligible for infertility criteria or not trying to conceive (PCOS)
27	Hmedeh C., et al. 2017 [S27]	Not relevant population	Ineligible for infertility criteria or not trying to conceive
28	Foroozanfard F. et al. 2017 [S28]	Not relevant population	Ineligible for infertility criteria or not trying to conceive (PCOS)
29	Azadi-Yazd M. et al. 2017 [S29]	Not relevant population	Ineligible for infertility criteria or not trying to conceive (PCOS)
30	Dokras A. et al. 2016 [S30]	Not relevant population	Ineligible for infertility criteria or not trying to conceive
31	Cooney L. et al. 2016 [S31]	Not relevant population	Ineligible for infertility criteria or not trying to conceive
32	Lai L.F. et al. 2015 [S32]	Not relevant population	Ineligible for infertility criteria or not trying to conceive (PCOS)

	Study	Category for exclusion	Detail Reason(s) for Exclusion
33	Sweatt K., et al. 2015 [S33]	Not relevant population	Ineligible for infertility criteria or not trying to conceive (PCOS)
34	Stepto N. 2015 [S34]	Not relevant population	Ineligible for infertility criteria or not trying to conceive (PCOS)
35	Yu L.L. et al. 2013 [S35]	Not relevant population	Ineligible for infertility criteria or not trying to conceive (PCOS)
36	Zello G.R. et al. 2013 [S36]	Not relevant population	Ineligible for infertility criteria or not trying to conceive (PCOS)
37	Thomson R.L. et al. 2013 [S37]	Not relevant population	Ineligible for infertility criteria or not trying to conceive, secondary analysis of cohort study
38	Jakubowicz D.D. and Wainstein Y.B. 2013 [S38]	Not relevant population	Ineligible for infertility criteria or not trying to conceive (PCOS)
39	Zheng Y. H. et al. 2013 [S39]	Not relevant population	Ineligible for infertility criteria or not trying to conceive
40	Nybacka Å . et al. 2013 [S40]	Not relevant population	Ineligible for infertility criteria or not trying to conceive
41	Thomson R. L. et al. 2012 [S41]	Not relevant population	Ineligible for infertility criteria or not trying to conceive, secondary analysis of RCT
42	Nyback A. et al. 2012a [S42]	Not relevant population	Ineligible for infertility criteria or not trying to conceive (PCOS)
43	Li D. 2012 [S43]	Not relevant population	Ineligible for infertility criteria or not trying to conceive (PCOS)
44	Le Donne M. A. et al. 2012 [S44]	Not relevant population	Ineligible for infertility criteria or not trying to conceive (PCOS)
45	Nyback A. et al. 2012b [S45]	Not relevant population	Ineligible for infertility criteria or not trying to conceive (PCOS)
46	Mehrabani H. H. et al. 2012 [S46]	Not relevant population	Ineligible for infertility criteria or not trying to conceive
47	Nybacka A. et al. 2011 [S47]	Not relevant population	Ineligible for infertility criteria or not trying to conceive (PCOS)
48	Moran L.J. et al. 2011 [S48]	Not relevant population	Ineligible for infertility criteria or not trying to conceive (PCOS)
49	Redman L.M. Elkind-Hirsch K. and Ravussin, E. 2011 [S49]	Not relevant population	Ineligible for infertility criteria or not trying to conceive, non-RCT

	Study	Category for exclusion	Detail Reason(s) for Exclusion
50	Moran L.J. et al. 2010 [S50]	Not relevant population	Ineligible for infertility criteria or not trying to conceive (PCOS)
51	Fux Otta C. et al. 2010 [S51]	Not relevant population	Ineligible for infertility criteria or not trying to conceive
52	Thomson R.L. et al. 2009 [S52]	Not relevant population	Ineligible for infertility criteria or not trying to conceive (PCOS)
53	Moro C.P., Elkind-Hirsch M. and Redman K.2009 [S53]	Not relevant population	Ineligible for infertility criteria or not trying to conceive (PCOS)
54	Panidis D. F. et al. 2008 [S54]	Not relevant population	Ineligible for infertility criteria or not trying to conceive (PCOS)
55	Thomson R.L. et al. 2008 [S55]	Not relevant population	Ineligible for infertility criteria or not trying to conceive
56	Florakis D. et al. 2008 [S56]	Not relevant population	Ineligible for infertility criteria or not trying to conceive (PCOS)
57	Bruner B.C. and Chizen, D.2006 [S57]	Not relevant population	Ineligible for infertility criteria or not trying to conceive (PCOS)
58	Gambiner A.P. et al. 2005 [S58]	Not relevant population	Ineligible for infertility criteria or not trying to conceive (PCOS)
59	Gambiner A.P. et al. 2004 [S59]	Not relevant population	Ineligible for infertility criteria or not trying to conceive (PCOS)
60	Vanky E., Salvesen K.A. and Carlsenm S. M. 2004 [S60]	Not relevant population	Ineligible for infertility criteria or not trying to conceive
61	Stamets K. et al. 2004 [S61]	Not relevant population	Ineligible for infertility criteria or not trying to conceive
62	Hoeger K. M. et al. 2004 [S62]	Not relevant population	Ineligible for infertility criteria or not trying to conceive
63	Gambineri A.P. et al. 2003 [S63]	Not relevant population	Ineligible for infertility criteria or not trying to conceive (PCOS)
64	Moran L. J. et al. 2003 [S64]	Not relevant population	Ineligible for infertility criteria or not trying to conceive
65	Pasquali et al. R. G. 2000 [S65]	Not relevant population	Ineligible for infertility criteria or not trying to conceive (PCOS)
66	Crave J. C. et al. 1995 [S66]	Not relevant population	Ineligible for infertility criteria or not trying to conceive
67	Guzick D. S. et al. 1994 [S67]	Not relevant population	Ineligible for infertility criteria or not trying to conceive

	Study	Category for exclusion	Detail Reason(s) for Exclusion
68	Pasquali R. F. and Venturoli R. 1986 [S68]	Not relevant population	Ineligible for infertility criteria or /not trying to conceive (PCOS)
69	Smith C.A. et al. 2018 [S69]	Not relevant population	Ineligible for obesity or overweight criteria,
70	Li J.W. et al. 2018 [S70]	Not relevant population	Ineligible for obesity or overweight criteria, secondary analysis of RCT
71	Wu X. K. et al. 2017 [S71]	Not relevant population	Ineligible for t obesity or overweight
72	Radin R.M. et al. 2016 [S72]	Not relevant population	Ineligible for obesity or overweight
73	Gillerman K. et al. 2016 [S73]	Not relevant population	Ineligible for obesity or overweight
74	Cochrane S. et al. 2016 [S74]	Not relevant population	Ineligible for obesity or overweight criteria
75	Firouzabadi R. D. et al. 2012 [S75]	Not relevant population	Ineligible for obesity or overweight criteria
76	Rosety M. Á . et al. 2017 [S 76]	Not relevant population	Infertility related to male
77	Maleki B. H. and Tartibian B. 2017 [S77]	Not relevant population	Infertility related to male
78	Fontenot G. K. and Podolski, J. 2017 [S78]	Not relevant population	Infertility related to male
79	Rafiee B.M. and Rahimi-Ghalati M. H. 2016 [S79]	Not relevant population	Infertility related to male
80	Collins C. E. et al. 2013 [S80]	Not relevant population	Infertility related to male
81	Machlitt, A.R., Steinheim, E. and Pfueller, B.F.2007 [S81]	Not relevant intervention	Pharmacological intervention
82	Steegers-Theunissen R. P. 2018 [S82]	Not relevant study design	Non-experimental study
83	Deng Y. et al. 2017 [S83]	Not relevant study design	Non-experimental study
84	Kaya Y. et al. 2016 [S84]	Not relevant study design	Non-RCT
85	Moore A.K.et al. 2016 [S85]	Not relevant study design	Non-RCT
86	Swaroop A. et al. 2015 [S86]	Not relevant study design	Non-RCT

	Study	Category for exclusion	Detail Reason(s) for Exclusion
87	Polotsky A.J.et al. 2015 [S87]	Not relevant study design	Secondary analysis of RCT
88	Miller P.B., Forstein D.A. and Styles S.2008 [S88]	Not relevant study design	Non-RCT
89	Palomba S.et al.2008 [S89]	Not relevant study design	Non-RCT
90	Tsagareli V., Noakes, M. and Norman R. J. 2006 [S90]	Not relevant study design	Non-RCT
91	Crosignani P. G. et al. 2003 [S91]	Not relevant study design	Non-RCT
92	Stener-Victorin E. et al. 2000 [S92]	Not relevant study design	Non-RCT
93	Clark A.M. et al. 1998 [S93]	Not relevant study design	Non-RCT
94	Galletly C. et al. 1996 [S94]	Not relevant study design	Non-RCT
95	Hollmann M. and Runnebaum B. Gerhard I.1996 [S95]	Not relevant study design	Non-RCT
96	Clark A.M. et al. 1996 [S96]	Not relevant study design	Non-RCT
97	Munir S. S. et al. 2018 [S97]	Excluded duplicates by hand	Same study as Munir S. S. et al. 2018 [S14]
98	Den Harink T. et al. 2019 [S98]	Excluded duplicates by hand	(Conference Abstract) Follow up study as Mutsaerts M.A. et al. 2016 [33]
99	Van Elten T. M. et al. 2019 [S99]	Excluded duplicates by hand	Same study as Mutsaerts M.A. et al. 2016 [33]
100	Wekker V. et al. 2018 [S100]	Excluded duplicates by hand	(Conference Abstract) Same study as Mutsaerts M.A. et al. 2016 [33]
101	Van Elten T. M. et al. 2018 [S101]	Excluded duplicates by hand	Same study as Mutsaerts M.A. et al. 2016 [33]
102	Einarsson S. et al. 2018 [S102]	Excluded duplicates by hand	(Conference Abstract) Same study as Einarsson S. et al. 2017 [26]
103	Wekker V. et al. 2017 [S103]	Excluded duplicates by hand	(Conference Abstract) Same study as Mutsaerts M.A. et al. 2016 [33]
104	Dammen L. et al. 2017 [S104]	Excluded duplicates by hand	(Conference Abstract) Same study as Mutsaerts M.A. et al. 2016 [33]
105	Arentz S. et al. 2017 [S105]	Excluded duplicates by hand	(Conference Abstract) Same study as Arentz S. et al.2017 [43]

	Study	Category for exclusion	Detail Reason(s) for Exclusion
106	Van Oers A. et al. 2016 [S106]	Excluded duplicates by hand	(Conference Abstract) Same study as Mutsaerts M.A. et al. 2016 [33]
107	Legro R. S. et al. 2016 [S107]	Excluded duplicates by hand	Same study as Legro R. S. et al. 2015 [39]
108	Legro R. S. et al.2015 [S108]	Excluded duplicates by hand	(Conference Abstract) Same study as Legro R. S. et al. 2015 [39]
109	Duval K. et al.2015 [S109)	Excluded duplicates by hand	(Conference abstract) same study as Belan M. et al. 2019 [28]
110	Belan M et al.2015 [S110)	Excluded duplicates by hand	(Conference abstract) same study as Belan M. et al. 2019 [31]
111	Van Oers A. et al.2014 [S111]	Excluded duplicates by hand	(Conference Abstract) Same study as Mutsaerts M.A. et al. 2016 [33]
112	Mutsaerts M.A.et al.2014 [S112]	Excluded duplicates by hand	(Conference Abstract) Same study as Mutsaerts M.A. et al. 2016 [33]
113	Legro R.S. et al. 2014 [S113]	Excluded duplicates by hand	(Conference Abstract) Same study as Legro R. S. et al. 2015 [39]
114	Sim K.A. et al. 2013 [S114]	Excluded duplicates by hand	(Conference Abstract) Same study as Sim K.A. et al. [37]
115	Jakubowicz D. et al. 2013 [S115]	Excluded duplicates by hand	Same study as Jakubowicz D.D. and Wainstein Y.B. 2013 [S 39]
116	Espinós-Gómez J.J. 2013 [S116]	Excluded duplicates by hand	Same study as Espinos J.J. et al. 2017 [32]
117	Sim K. Denyer G. and Caterson I. 2012 [S117]	Excluded duplicates by hand	(Conference Abstract) Same study as Sim K.A. et al. [37]
118	Kjellberg A.T. 2012 [S118]	Excluded duplicates by hand	Same study as Einarsson S. et al.2017 [26]
119	Ma H. and Zheng Y.H. 2012 [S119]	Excluded duplicates by hand	Same study as Zheng Y. H. et al. 2013 [S39]
120	Palomba S. et al. 2010 [S120)	Excluded duplicates by hand	(Conference Abstract) Same study as Palomba S. et al. 2010. [40]
121	Yin Y. et al. 2018 [S121]	Not available results in English	Not available results in English
122	Shen L.Y. et al. 2018 [S122]	Not available results in English	Not available results in English
123	Cao Y. et al. 2017 [S123]	Not available results in English	Not available results in English

	Study	Category for exclusion	Detail Reason(s) for Exclusion
124	Nasrekani Z. A. and Fathi M. 2016 [S124]	Not available results in English	Not available results in English
125	Lai M. H. et al. 2010 [S125]	Not available results in English	Not available results in English
126	Hamayeli Mehrabani H. et al. 2010 [S126]	Not available results in English	Not available results in English
127	Esmaeilzadeh S. et al. 2010 [S127]	Not available results in English	Not available results in English
128	Baillargeon J.P. 2019 [S128]	Protocol only study	Protocol study (ongoing study) , same study as Belan M. et al. 2019 [31]
129	Casals G. 2018 [S129]	Protocol only study	Protocol study (ongoing study)
130	Sathyapalan T. 2017 [S130]	Protocol only study	Protocol study
131	Jiskoot G. et al. 2017 [S131	Protocol only study	Protocol study, same study as Jiskoot G. et al.2019 [35]
132	Duval K. et al. 2015 a [S132]	Protocol only study	Protocol study, same study as Belan M. et al. 2019 [31]
133	Santoro N. et al. 2015 [S133]	Protocol only study	Protocol study (ongoing study)
134	Mutsaerts M.A. et al. 2010 [S134]	Protocol only study	Protocol study, as Mutsaerts M.A. et al. 2016 [33]
Excluded during data extraction due to sharing same population			
135	van Elten T.M. et al. 2019 [S135]	Sharing same population	Follow up study as Mutsaerts M.A. et al. 2016 [33]
136	van Dammen L. et al. 2019 [S136]	Sharing same population	Follow up study as Mutsaerts M.A. et al. 2016 [33]
137	Einarsson S. et al. 2019 [S137]	Sharing same population	Secondary analysis of Einarsson S. et al.2017 [26]
138	van Elten T.M. et al. 2018 [S138]	Sharing same population	Secondary analysis as Mutsaerts M.A. et al. 2016 [33]
139	Wekker V. et al. 2018 [S139]	Sharing same population	Follow up study as Mutsaerts M.A. et al. 2016 [33]
140	van Dammen L. et al. 2018 [S140]	Sharing same population	Secondary analysis as Mutsaerts M.A. et al. 2016 [33]
141	van Oers A.M. et al. 2017 [S141]	Sharing same population	Cost effective analysis as Mutsaerts M.A. et al. 2016 [33]
142	van Oers A.M. et al. 2016 [S142]	Sharing same population	Subgroup analysis as Mutsaerts M.A. et al. 2016 [33]

	Study	Category for exclusion	Detail Reason(s) for Exclusion
143	Moran L.J. et al. 2016 [S143]	Sharing same population	Secondary analysis as Moran L. et al. 2011[29]
144	Jiskoot G. et al. 2018 [S144]	Sharing same population	(Abstract only) same study as Jiskoot G. et al.2019 [35]
145	Belan M. et al. 2019 a [S145]	Sharing same population	(Conference abstract) same study as Belan M. et al. 2019 [31]
146	Belan M.et al. 2019b [S146]	Sharing same population	(Conference abstract) same study as Belan M. et al. 2019 [31]
147	Duval K. et al. 2015b [S147]	Sharing same population	(Conference abstract) same study as Belan M. et al. 2019 [31]

References

S1. Kadoura, S. A., M. Nattouf, A. H., Effect of calcium and vitamin d supplements as an adjuvant therapy to metformin on menstrual cycle abnormalities, hormonal profile, and IGF-1 system in polycystic ovary syndrome patients: A randomized, placebo-controlled clinical trial. *Adv Pharmacol Sci* **2019,** *2019*. https://doi.org/10.1155/2019/9680390

S2. Jamilian, M. Z. M., S. Amiri Siavashani, M. Karimi, M. Mafi, A. Ostadmohammadi, V. Asemi, Z., The Influences of Chromium Supplementation on Glycemic Control, Markers of Cardio-Metabolic Risk, and Oxidative Stress in Infertile Polycystic ovary Syndrome Women Candidate for In vitro Fertilization: a Randomized, Double-Blind, Placebo-Controlled Trial. *Biological Trace Element Research* **2018,** *185* (1), 48-55. https://doi.org/10.1007/s12011-017-1236-3

S3. Vizza, L. S., C. A. Swaraj, S. Agho, K. Cheema, B. S., The feasibility of progressive resistance training in women with polycystic ovary syndrome: A pilot randomized controlled trial. *BMC Sports Sci Med Rehabil* **2016,** *8* (1). https://doi.org/10.1186/s13102-016-0039-8

S4. Almenning, I.; Rieber-Mohn, A.; Lundgren, K. M.; Lovvik, T. S.; Garnaes, K. K.; Moholdt, T., Effects of high intensity interval training and strength training on metabolic, cardiovascular and hormonal outcomes in women with polycystic ovary syndrome: a pilot study. *PloS ONE* **2015,** *10* (9) https://doi.org/10.1371/journal.pone.0138793

S5. Glueck, C. J.; Goldenberg, N.; Pranikoff, J.; Khan, Z.; Padda, J.; Wang, P., Effects of metformin-diet intervention before and throughout pregnancy on obstetric and neonatal outcomes in patients with polycystic ovary syndrome. *Curr Med Res Opin* **2013,** *29* (1), 55-62. https://doi.org/10.1185/03007995.2012.755121

S6. Johansson, J.; Redman, L.; Veldhuis, P. P.; Sazonova, A.; Labrie, F.; Holm, G.; Johannsson, G.; Stener-Victorin, E., Acupuncture for ovulation induction in polycystic ovary syndrome: a randomized controlled trial. *Am. J. Physiol. Endocrinol. Metab.* **2013,** *304* (9), E934-943. https://doi.org/10.1152/ajpendo.00039.2013

S7. Jedel, E. L., F. Oden, A. Holm, G. Nilsson, L. Janson, P. O. Lind, A. K. Ohlsson, C. Stener-Victorin, E., Impact of electro-acupuncture and physical exercise on hyperandrogenism and oligo/amenorrhea in women with polycystic ovary syndrome: a randomized controlled trial. *Am. J. Physiol. Endocrinol. Metab.* **2011,** *300* (1), E37-E45. https://doi.org/10.1152/ajpendo.00495.2010

S8. Wehr, E. P., T. R. Obermayer-Pietsch, B., Effect of vitamin D3 treatment on glucose metabolism and menstrual frequency in polycystic ovary syndrome women: A pilot study. *J. Endocrinol. Invest.* **2011,** *34* (10), 757-763. https://doi.org/10.3275/7748

S9. Moran, L. J.; Noakes, M.; Clifton, P. M.; Wittert, G. A.; Williams, G.; Norman, R. J., Short-term meal replacements followed by dietary macronutrient restriction enhance weight loss in polycystic ovary syndrome. *Am. J. Clin. Nutr.* **2006,** *84* (1), 77-87. https://doi.org/10.1093/ajcn/84.1.77

S10. Esmael, M. E. H. A., G. A. Nashed, A. B., Effect of pulsed electromagnetic field versus aerobic exercises on women with polycystic ovary syndrome: A single-blind randomized controlled trial. *ACAM* **2019,** *10* (6), 676-681. https://doi.org/10.4328/ACAM.6101

S11. Donne, M. L. E. M., D. Alibrandi, A. Papa, M. Benvenga, S., Effects of three treatment modalities (diet, myoinositol or myoinositol associated with D-chiro-inositol) on clinical and body composition outcomes in women with polycystic ovary syndrome. *Eur Rev Med Pharmacol Sci* **2019,** *23* (5), 2293-2301. https://doi.org/10.26355/eurrev_201903_17278
S12. Tiwari, N.; Pasrija, S.; Jain, S., Randomised controlled trial to study the efficacy of exercise with and without metformin on women with polycystic ovary syndrome. *Eur. J. Obstet. Gynecol. Reprod. Biol.* **2019,** *234*, 149-154. https://doi.org/10.1016/j.ejogrb.2018.12.021
S13. Hiam, D.; Patten, R.; Gibson-Helm, M.; Moreno-Asso, A.; McIlvenna, L.; Levinger, I.; Harrison, C.; Moran, L. J.; Joham, A.; Parker, A.; et al., The effectiveness of high intensity intermittent training on metabolic, reproductive and mental health in women with polycystic ovary syndrome: study protocol for the iHIT-randomised controlled trial. *Trials* **2019,** *20 (1)* 221. https://doi.org/10.1186/s13063-019-3313-8
S14. Munir, S. S. J., S. Alvi, N. Khan, D. Bin Younis, B. Khan, M., Role of Orlistat Combined With Life Style in the Management of Obese Patients with Polycystic Ovarian Syndrome. *PJMHS* **2018,** *12* (2), 477-480.
S15. Abd Al Samea, G. A. M., N. F. Hamada, H. A. Gabr, A. A., Influence of pulsed electromagnetic field on dermatological symptoms of hyperandrogen in obese women with polycystic ovarian syndrome: Influence of pulsed electromagnetic field on dermatological symptoms of hyperandrogen in obese women. *Journal of Clinical and Analytical Medicine* **2018,** *9* (6), 493-497. https://doi.org/10.4328/JCAM.5816
S16. Miranda-Furtado, C. L.; Ribeiro, V. B.; Lopes, I. P.; Silva, R. C.; Kogure, G. S.; Pedroso, D. C.; Ferriani, R. A.; Reis, R. M. In *Aerobic physical training reduces anthropometric indexes and hyperandrogenism in polycystic ovary syndrome*, 74th annual congress of the american society for reproductive medicine(ASRM 2018), Denver colorado, united states, 2018; Fertil. Steril.: Denver colorado, united states, 2018; p e9.
S17. Mani, H.; Chudasama, Y.; Hadjiconstantinou, M.; Bodicoat, D. H.; Edwardson, C.; Levy, M. J.; Gray, L. J.; Barnett, J.; Daly, H.; Howlett, T. A.; et al., Structured education programme for women with polycystic ovary syndrome: a randomised controlled trial. *Endocr Connect* **2018,** *7* (1), 26-35. https://doi.org/10.1530/EC-17-0274
S18. Lopes, I. P.; Ribeiro, V. B.; Reis, R. M.; Silva, R. C.; Dutra de Souza, H. C.; Kogure, G. S.; Ferriani, R. A.; Silva Lara, L. A. D., Comparison of the Effect of Intermittent and Continuous Aerobic Physical Training on Sexual Function of Women With Polycystic Ovary Syndrome: randomized Controlled Trial. *J Sex Med* **2018,** *15* (11), 1609-1619.
S19. Lara, L.; Lopes, I. P.; Dos Reis, R. M.; Ribeiro, V. B.; De Souza, H. C. D.; Silva, R. C., Aerobic physical training improves sexual function and qol of pcos women: randomized conrrolled tria. *Int J Gynaecol Obstet* **2018,** *143*, 357-358. https://doi.org/10.1002/ijgo.12582
S20. Khan, A. A.; Begum, W., Efficacy of Darchini in the management of polycystic ovarian syndrome: a randomized clinical study. *Journal of herbal medicine* **2018**.
S21. Foroozanfard, F.; Rafiei, H.; Samimi, M.; Gilasi, H. R.; Gorjizadeh, R.; Heidar, Z.; Asemi, Z., The effects of dietary approaches to stop hypertension diet on weight loss, anti-Müllerian hormone and metabolic profiles in women with polycystic ovary syndrome: a randomized clinical trial. *Clin. Endocrinol.* **2017,** *87* (1), 51-58.
S22. Matsuzaki, T. D., T. Oki, T. Ishihara, O. Okagaki, R. Kajihara, T. Tamura, M. Kotsuji, F. Tajima, K. Kawano, M. Ishizuka, B. Irahara, M., Weight reduction by using a formula diet recovers menstruation in obese patients with an ovulatory disorder. *Reproductive Medicine and Biology* **2017**.
S23. Bauer, J. L. A.-S., Z. Malkhasyan, A. Harris, M. Eckel, R. H. Bradford, A. P. Robledo, C. Gee, N. A. Polotsky, A. J. In *Omega-3 fatty acid supplementation improves luteal function in obese women*, 99th Annual Meeting of the Endocrine Society, ENDO 2017, United States, Orlando, FL, April 2017; Endocr. Rev.: United States, Orlando, FL, 2017.
S24. McBreairty, L. E.; Kazemi, M.; Gordon, J. J.; Pierson, R. A.; Chizen, D. R.; Chilibeck, P. D.; Zello, G. A., A randomized clinical trial in women with polycystic ovary syndrome: effects of a pulse-based diet and exercise intervention on blood lipids, body composition and reproductive measures. *FASEB journal* **2017,** *31* (1 Supplement 1).
S25. Lai, L.; Flower, A.; Prescott, P.; Wing, T.; Moore, M.; Lewith, G., Standardised versus individualised multiherb Chinese herbal medicine for oligomenorrhoea and amenorrhoea in polycystic ovary syndrome: a randomised feasibility and pilot study in the UK. *BMJ open* **2017,** *7* (2), e011709.
S26. Jafari-Sfidvajani, S.; Ahangari, R.; Hozoori, M.; Mozaffari-Khosravi, H.; Fallahzadeh, H.; Nadjarzadeh, A., The effect of vitamin D supplementation in combination with low-calorie diet on anthropometric indices and androgen hormones in women with polycystic ovary syndrome: a double-blind, randomized, placebo-controlled trial. *J. Endocrinol. Invest.* **2017**, 1-11.

S27. Hmedeh, C.; Ghazeeri, G.; Tinworth, L.; Tewfik, I. In *The effect of 6 months weight -loss/maintenance on anthropometric, biochemical and psychological profile in Lebanese PCOS women: a prospective randomised control study*, 33rd Annual Meeting of the European Society of Human Reproduction and Embryology., Geneva. Switzerland., July 2017; Hum. Reprod. : Geneva. Switzerland., 2017; pp i451-i452.

S28. Foroozanfard, F.; Rafiei, H.; Samimi, M.; Gilasi, H. R.; Gorjizadeh, R.; Heidar, Z.; Asemi, Z., The effects of dietary approaches to stop hypertension diet on weight loss, anti-Mullerian hormone and metabolic profiles in women with polycystic ovary syndrome: A randomized clinical trial. *Clin. Endocrinol.* **2017,** *87* (1), 51-58.

S29. Azadi-Yazdi, M.; Karimi-Zarchi, M.; Salehi-Abargouei, A.; Fallahzadeh, H.; Nadjarzadeh, A., Effects of Dietary Approach to Stop Hypertension diet on androgens, antioxidant status and body composition in overweight and obese women with polycystic ovary syndrome: a randomised controlled trial. *J Hum Nutr Diet* **2017,** *30* (3), 275-283.

S30. Dokras, A.; Sarwer, D. B.; Allison, K. C.; Milman, L.; Kris-Etherton, P. M.; Kunselman, A. R.; Stetter, C. M.; Williams, N. I.; Gnatuk, C. L.; Estes, S. J.; et al., Weight Loss and Lowering Androgens Predict Improvements in Health-Related Quality of Life in Women With PCOS. *Int J Clin Endocrinol Metab* **2016,** *101* (8), 2966-2974.

S31. Cooney, L.; Milman, L. W.; Sammel, M.; Allison, K.; Epperson, C.; Dokras, A. In *Cognitive behavioral therapy improves weight loss and quality of life in women with polycystic ovary syndrome (PCOS)*, ASRM Scientific Congress and Expo Scaling New Heights in Reproductive Medicine, ASRM 2016., Salt Lake City, UT. United States, Oct,2016; Fertil. Steril.: Salt Lake City, UT. United States, 2016; pp e252-e253.

S32. Lai, L. F., A. Prescott, P. Moore, M. Lewith, G., Chinese herbal medicine for oligomenorrhoea and amenorrhoea in polycystic ovary syndrome: A randomised feasibility study in the United Kingdom. *Integr Med Res* **2015,** *4* (1), 15.

S33. Sweatt, K.; Ovalle, F.; Azziz, R.; Gower, B. In *The effect of diet and exercise in women with polycystic ovary syndrome*, Experimental Biology 2015, EB Boston, MA United States., March2015; FASEB J.
: EB Boston, MA United States., 2015.

S34. N., S. The (i)mpact of (H)igh (I)ntensity intermittent (T)raining on health and mechanisms of insulin resistance in women with (P)oly(c)ystic (O)vary (S)yndrome: the iHIT-PCOS randomised control trial. https://www.cochranelibrary.com/central/doi/10.1002/central/CN-01842636/full (accessed 20 Sept.).

S35. Yu, L.; Liao, Y.; Wu, H.; Zhao, J.; Wu, L.; Shi, Y.; Fang, J., Effects of electroacupuncture and Chinese kidney-nourishing medicine on polycystic ovary syndrome in obese patients. *J Tradit Chin Med* **2013,** *33* (3), 287-93.

S36. Zello, G. R., K. Rooke, J. J. Serrao, S. Chizen, D. R. Chilibeck, P. D., A pulse-based diet intervention in women with polycystic ovarian syndrome (PCOS): Compliance, and effects on body composition, fertility and metabolic measures. *FASEB J.* **2013,** *27.*

S37. Thomson, R. L. S., S. Brinkworth, G. D. Noakes, M. Buckley, J. D., Seasonal effects on vitamin D status influence outcomes of lifestyle intervention in overweight and obese women with polycystic ovary syndrome. *Fertil. Steril.* **2013,** *99* (6), 1779-1785.

S38. Jakubowicz, D. D., Y. B. Wainstein, J., The influence of meal timing on glucose metabolism and hyperandrogenism in lean women with polycystic ovary syndrome. *Diabetes* **2013,** *62,* A399.

S39. Zheng, Y. H.; Wang, X. H.; Lai, M. H.; Yao, H.; Liu, H.; Ma, H. X., Effectiveness of abdominal acupuncture for patients with obesity-type polycystic ovary syndrome: a randomized controlled trial. *J Altern Complement Med* **2013,** *19* (9), 740-745.

S40. Nybacka, Å.; Carlström, K.; Fabri, F.; Hellström, P. M.; Hirschberg, A. L., Serum antimüllerian hormone in response to dietary management and/or physical exercise in overweight/obese women with polycystic ovary syndrome: secondary analysis of a randomized controlled trial. *Fertil. Steril.* **2013,** *100* (4), 1096-1102.

S41. Thomson, R. L. B., G. D. Noakes, M. Clifton, P. M. Norman, R. J. Buckley, J. D., The effect of diet and exercise on markers of endothelial function in overweight and obese women with polycystic ovary syndrome. *Hum. Reprod.* **2012,** *27* (7), 2169-2176.

S42. Nyback, A.; Fabri, F.; Hellstrom, P. M.; Hirschberg, A. L.; Stahle, A., Plasma Anti-Mullerian Hormone (AMH) levels correlate and predict the response to lifestyle intervention in polycystic ovarian syndrome. *Clinical nutrition, supplement* **2012,** *7* (1), 255.

S43. Li, D., The effect of acupuncture on ovulatory frequency and pituitary gonadotropin hormones in women with polycystic ovarian syndrome. *Focus Altern Complement Ther* **2012,** *17* (2), 121-122.

S44. Le Donne, M. A., A. Giarrusso, R. Lo Monaco, I. Muraca, U., Diet, metformin and inositol in overweight and obese women with polycystic ovary syndrome: Effects on body composition. *Minerva Ginecol* **2012,** *64* (1), 23-29.
S45. Nyback, A. F., F. Hellström, P. M. Hirschberg, A. L. Sta˚hle, A., Plasma Anti-Müllerian Hormone (AMH) levels correlate and predict the response to lifestyle intervention in polycystic ovarian syndrome. *Clinical Nutrition, Supplement* **2012,** *7* (1), 255.
S46. Mehrabani, H. H.; Salehpour, S.; Amiri, Z.; Farahani, S. J.; Meyer, B. J.; Tahbaz, F., Beneficial effects of a high-protein, low-glycemic-load hypocaloric diet in overweight and obese women with polycystic ovary syndrome: a randomized controlled intervention study. *J Am Coll Nutr* **2012,** *31* (2), 117-125.
S47. Nybacka, A. C., K. Stahle, A. Nyren, S. Hellstrom, P. M. Hirschberg, A. L., Randomized comparison of the influence of dietary management and/or physical exercise on ovarian function and metabolic parameters in overweight women with polycystic ovary syndrome. *Fertil. Steril.* **2011,** *96* (6), 1508-1513.
S48. Moran, L. J.; Harrison, C. L.; Hutchison, S. K.; Stepto, N. K.; Strauss, B. J.; Teede, H. J., Exercise decreases anti-mullerian hormone in anovulatory overweight women with polycystic ovary syndrome: a pilot study. *Horm. Metab. Res.* **2011,** *43* (13), 977-9.
S49. Redman, L. M. E.-H., K. Ravussin, E., Aerobic exercise in women with polycystic ovary syndrome improves ovarian morphology independent of changes in body composition. *Fertil. Steril.* **2011,** *95* (8), 2696-2699.
S50. Moran, L. J.; Noakes, M.; Clifton, P. M.; Norman, R. J., The effect of modifying dietary protein and carbohydrate in weight loss on arterial compliance and postprandial lipidemia in overweight women with polycystic ovary syndrome. *Fertil. Steril.* **2010,** *94* (6), 2451-4.
S51. Fux Otta, C.; Wior, M.; Iraci, G. S.; Kaplan, R.; Torres, D.; Gaido, M. I.; Wyse, E. P., Clinical, metabolic, and endocrine parameters in response to metformin and lifestyle intervention in women with polycystic ovary syndrome: a randomized, double-blind, and placebo control trial. *Gynecol. Endocrinol.* **2010,** *26* (3), 173-178.
S52. Thomson, R. L.; Buckley, J. D.; Moran, L. J.; Noakes, M.; Clifton, P. M.; Norman, R. J.; Brinkworth, G. D., The effect of weight loss on anti-Mullerian hormone levels in overweight and obese women with polycystic ovary syndrome and reproductive impairment. *Hum. Reprod.* **2009,** *24* (8), 1976-81.
S53. Moro, C.; Pasarica, M.; Elkind-Hirsch, K.; Redman, L. M., Aerobic exercise training improves atrial natriuretic peptide and catecholamine-mediated lipolysis in obese women with polycystic ovary syndrome. *J. Clin. Endocrinol. Metab.* **2009,** *94* (7), 2579-86.
S54. Panidis, D.; Farmakiotis, D.; Rousso, D.; Kourtis, A.; Katsikis, I.; Krassas, G., Obesity, weight loss, and the polycystic ovary syndrome: effect of treatment with diet and orlistat for 24 weeks on insulin resistance and androgen levels. *Fertil. Steril.* **2008,** *89* (4), 899-906.
S55. Thomson, R. L.; Buckley, J. D.; Noakes, M.; Clifton, P. M.; Norman, R. J.; Brinkworth, G. D., The effect of a hypocaloric diet with and without exercise training on body composition, cardiometabolic risk profile, and reproductive function in overweight and obese women with polycystic ovary syndrome. *J. Clin. Endocrinol. Metab.* **2008,** *93* (9), 3373-3380.
S56. D Florakis , E. D.-K., I Katsikis , GP Nassis , A Karkanaki , N Georgopoulos and D Panidis Effect of Diet Plus Sibutramine on Hormonal and Metabolic Features in Overweight and Obese Women With PCOS. https://www.cochranelibrary.com/central/doi/10.1002/central/CN-01514993/full (accessed 20, Sept).
S57. Bruner, B. C., K. Chizen, D., Effects of exercise and nutritional counseling in women with polycystic ovary syndrome. *Appl Physiol Nutr Metab* **2006,** *31* (4), 384-391.
S58. Gambineri, A. P., L. De Iasio, R. Cantelli, B. Cognini, G. E. Filicori, M. Barreca, A. Diamanti-Kandarakis, E. Pagotto, U. Pasquali, R., Efficacy of octreotide-LAR in dieting women with abdominal obesity and polycystic ovary syndrome. *J. Clin. Endocrinol. Metab.* **2005,** *90* (7), 3854-3862.
S59. Gambineri, A. P., C. Genghini, S. Morselli-Labate, A. M. Cacciari, M. Pagotto, U. Pasquali, R., Effect of flutamide and metformin administered alone or in combination in dieting obese women with polycystic ovary syndrome. *Clin. Endocrinol.* **2004,** *60* (2), 241-249.
S60. Vanky, E.; Salvesen, K.; Carlsen, S. J. H. R., Six-month treatment with low-dose dexamethasone further reduces androgen levels in PCOS women treated with diet and lifestyle advice, and metformin. *Hum. Reprod.* **2004,** *19* (3), 529-533.
S61. Stamets, K.; Taylor, D. S.; Kunselman, A.; Demers, L. M.; Pelkman, C. L.; Legro, R. S., A randomized trial of the effects of two types of short-term hypocaloric diets on weight loss in women with polycystic ovary syndrome. *Fertil. Steril.* **2004,** *81* (3), 630-637.
S62. Hoeger, K. M.; Kochman, L.; Wixom, N.; Craig, K.; Miller, R. K.; Guzick, D. S., A randomized, 48-week, placebo-controlled trial of intensive lifestyle modification and/or metformin therapy in overweight women with polycystic ovary syndrome: a pilot study. *Fertil. Steril.* **2004,** *82* (2), 421-429.

S63. Gambineri, A. P., U. Tschöp, M. Vicennati, V. Manicardi, E. Carcello, A. Cacciari, M. De Iasio, R. Pasquali, R., Anti-androgen treatment increases circulating ghrelin levels in obese women with polycystic ovary syndrome. *J. Endocrinol. Invest.* **2003,** *26* (7), 629-634.

S64. Moran, L. J.; Noakes, M.; Clifton, P. M.; Tomlinson, L.; Galletly, C.; Norman, R. J., Dietary composition in restoring reproductive and metabolic physiology in overweight women with polycystic ovary syndrome. *J. Clin. Endocrinol. Metab.* **2003,** *88* (2), 812-819.

S65. Pasquali, R.; Gambineri, A.; Biscotti, D.; Vicennati, V.; Gagliardi, L.; Colitta, D.; Fiorini, S.; Cognigni, G. E.; Filicori, M.; Morselli-Labate, A. M., Effect of long-term treatment with metformin added to hypocaloric diet on body composition, fat distribution, and androgen and insulin levels in abdominally obese women with and without the polycystic ovary syndrome. *J. Clin. Endocrinol. Metab.* **2000,** *85* (8), 2767-74.

S66. Crave, J. C.; Fimbel, S.; Lejeune, H.; Cugnardey, N.; Déchaud, H.; Pugeat, M., Effects of diet and metformin administration on sex hormone-binding globulin, androgens, and insulin in hirsute and obese women. *J. Clin. Endocrinol. Metab.* **1995,** *80* (7), 2057-2062.

S67. Guzick, D. S.; Wing, R.; Smith, D.; Berga, S. L.; Winters, S. J., Endocrine consequences of weight loss in obese, hyperandrogenic, anovulatory women. *Fertil. Steril.* **1994,** *61* (4), 598-604.

S68. Pasquali, R. F., R. Venturoli, S., Effect of weight loss and antiandrogenic therapy on sex hormone blood levels and insulin resistance in obese patients with polycystic ovaries. *Am. J. Obstet. Gynecol.* **1986,** *154* (1), 139-144.

S69. Smith, C. A. D. L., S. Chapman, M. Ratcliffe, J. Norman, R. J. Johnson, N. P. Boothroyd, C. Fahey, P., Effect of acupuncture vs sham acupuncture on live births among women undergoing in vitro fertilization: A randomized clinical trial. *Obstet Gynecol Surv* **2018,** *73* (8), 466-467.

S70. Li, J. W., Q. Wu, X. K. Zhou, Z. M. Fu, P. Chen, X. H. Yan, Y. Wang, X. Yang, Z. W. Li, W. L. Stener-Victorin, E. Legro, R. S. Ng, E. H. Y. Zhang, H. Mol, B. W. J. Wang, C. C., Effect of exposure to second-hand smoke from husbands on biochemical hyperandrogenism, metabolic syndrome and conception rates in women with polycystic ovary syndrome undergoing ovulation induction. *Hum. Reprod.* **2018,** *33* (4), 617-625.

S71. Wu, X. K.; Stener-Victorin, E.; Kuang, H. Y.; Ma, H. L.; Gao, J. S.; Xie, L. Z.; Hou, L. H.; Hu, Z. X.; Shao, X. G.; Ge, J.; et al., Effect of acupuncture and clomiphene in Chinese women with polycystic ovary syndrome: a randomized clinical trial. *JAMA - journal of the american medical association* **2017,** *317* (24), 2502-2514.

S72. Radin, R. M., S. L. Silver, R. M. Lynch, A. M. Perkins, N. Sjaarda, L. Schisterman, E., Recent weight-control efforts before trying to conceive, fecundability, and ovulation among fecund women. *Fertil. Steril.* **2016,** *106*, e102.

S73. Gillerman, K.; Rehman, N.; Dilgil, M.; Homburg, R. In *The impact of acupuncture on IVF success rates: a randomized controlled trial*, 32nd Annual Meeting of the European Society of Human Reproduction and Embryology, Helsinki,Filand, July,2016; Hum. Reprod. : Helsinki,Filand, 2016; pp i89-.

S74. Cochrane, S.; Smith, C. A.; Possamai-Inesedy, A.; Bensoussan, A., Prior to Conception: the Role of an Acupuncture Protocol in Improving Women's Reproductive Functioning Assessed by a Pilot Pragmatic Randomised Controlled Trial. *Evid Based Complement Alternat Med* **2016,** *2016*.

S75. Firouzabadi, R. D. A., A. Modarresi, S. Sekhavat, L. MohammadTaheri, S., Therapeutic effects of calcium & vitamin D supplementation in women with PCOS. *Complement Ther Clin Pract* **2012,** *18* (2), 85-88.

S76. Rosety, M. Á. D., A. J. Rosety, J. M. Pery, M. T. Brenes-Martín, F. Bernardi, M. García, N. Rosety-Rodríguez, M. Ordoñez, F. J. Rosety, I., Exercise improved semen quality and reproductive hormone levels in sedentary obese adults. *Nutricion hospitalaria* **2017,** *34* (3), 603-607.

S77. Maleki, B. H.; Tartibian, B., High-Intensity Exercise Training for Improving Reproductive Function in Infertile Patients: a Randomized Controlled Trial. *Journal of obstetrics and gynaecology canada* **2017,** *39* (7), 545-558.

S78. Fontenot, G. K. T., J. Podolski, J. In *Diet and exercise results in an increased testosterone, while addition of enclomiphene citrate significantly increases free-testosterone and improves lean body mass*, 99th Annual Meeting of the Endocrine Society (ENDO 2017), United States, Orlando, FL, April, 2017; Endocr. Rev.: United States, Orlando, FL, 2017.

S79. Rafiee, B. M., M. H. Rahimi-Ghalati, N., Comparing the Effectiveness of Dietary Vitamin C and Exercise Interventions on Fertility Parameters in Normal Obese Men. *Urol J* **2016,** *13* (2), 2635-2639.

80. Collins, C. E.; Jensen, M. E.; Young, M. D.; Callister, R.; Plotnikoff, R. C.; Morgan, P. J., Improvement in erectile function following weight loss in obese men: the SHED-IT randomized controlled trial. *Obes Res Clin Pract* **2013,** *7* (6), e450-4.

S81. Machlitt, A. R.; Steinheim, E.; Pfueller, B. F., Effects of metformin plus lifestyle modification on menstrual cycle and body mass index are not influenced by insulin resistance: a 16 week, double-blind, controlled, randomised study in PCOS. *Fertil. Steril.* **2007,** *88 Suppl 1*, 15-16, Abstract no: 41.

82. Steegers-Theunissen, R. P. M. In *Preconceptional personalised mHealth lifestyle coaching:\rFirst results of a randomized controlled trial in couples undergoing IVF/ICSI treatment*, the 34rd Annual Meeting of the European Society of Human Reproduction and Embryology, Bercelona,Spain, July, 2018; Hum. Reprod.: Bercelona,Spain, 2018; pp i74-i75.
S83. Deng, Y.; Xie, L.; Wu, X. K.; Ng, E. H.; Victorin, E. S.; Zhang, H.; Legro, R. S.; Mol, B. W. In *Prediction models for ovulation, conception, pregnancy and live birth in infertile women with polycystic ovary syndrome*, 33rd Annual Meeting of the European Society of Human Reproduction and Embryology. , Geneva. Switzerland, July,2017; Hum. Reprod.: Geneva. Switzerland, 2017; pp i454-i455.
S84. Kaya, Y.; Kizilkaya Beji, N.; Aydin, Y.; Hassa, H., The effect of health-promoting lifestyle education on the treatment of unexplained female infertility. *Eur. J. Obstet. Gynecol. Reprod. Biol.* **2016,** *207*, 109-114.
S85. Moore, A. K. R., R. Sandberg, J. Holt-Lunstad, J. Carrell, D. T. Straseski, J. Peterson, C. M. Johnstone, E. B. In *Intensive lifestyle intervention including emotionally-focused couples therapy leads to more pregnancies and weight loss in obese infertile couples*, ASRM Scientific Congress and Expo Scaling New Heights in Reproductive Medicine(ASRM 2016), Salt Lake City, UT,United States, , Oct.2016; Fertil. Steril.: Salt Lake City, UT,United States, , 2016; p e101.
S86. Swaroop, A.; Jaipuriar, A. S.; Gupta, S. K.; Bagchi, M.; Kumar, P.; Preuss, H. G.; Bagchi, D., Efficacy of a Novel Fenugreek Seed Extract (Trigonella foenum-graecum, Furocyst) in Polycystic Ovary Syndrome (PCOS). *Int J Med Sci* **2015,** *12* (10), 825-831.
S87. Polotsky, A. J.; Allshouse, A. A.; Krawetz, S.; Santoro, N.; Eisenberg, E.; Zhang, H.; Diamond, M. P. In *Male obesity is an independent risk factor for treatment failure among couples with unexplained infertility in the assessment of multiple intrauterine gestations from ovarian stimulation (AMIGOS) trial*, 71st Annual Meeting of the American Society for Reproductive Medicine(ASRM 2015), Baltimore, MD United States., Oct. 2015 Fertil. Steril.: Baltimore, MD United States., 2015; p e78.
S88. Miller, P. B.; Forstein, D. A.; Styles, S., Effect of short-term diet and exercise on hormone levels and menses in obese, infertile women. *J Reprod Med* **2008,** *53* (5), 315-9.
S89. Palomba, S. G., F. Falbo, A. Russo, T. Oppedisano, R. Tolino, A. Colao, A. Vigorito, C. Zullo, F. Orio, F., Structured exercise training programme versus hypocaloric hyperproteic diet in obese polycystic ovary syndrome patients with anovulatory infertility: A 24-week pilot study. *Hum. Reprod.* **2008,** *23* (3), 642-650.
S90. Tsagareli, V.; Noakes, M.; Norman, R. J., Effect of a very-low-calorie diet on in vitro fertilization outcomes. *Fertil. Steril.* **2006,** *86* (1), 227-9.
S91. Crosignani, P. G. C., M. Vegetti, W. Somigliana, E. Gessati, A. Ragni, G., Overweight and obese anovulatory patients with polycystic ovaries: Parallel improvements in anthropometric indices, ovarian physiology and fertility rate induced by diet. *Hum. Reprod.* **2003,** *18* (9), 1928-1932.
S92. Stener-Victorin, E. W., U. Tägnfors, U. Lundeberg, T. Lindstedt, G. Janson, P. O., Effects of electro-acupuncture on anovulation in women with polycystic ovary syndrome. *Acta Obstet Gynecol Scand* **2000,** *79* (3), 180-188.
S93. Clark, A. M. T., B. Tomlinson, L. Galletley, C. Norman, R. J., Weight loss in obese infertile women results in improvement in reproductive outcome for all forms of fertility treatment. *Hum. Reprod.* **1998,** *13* (6), 1502-1505.
S94. Galletly, C.; Clark, A.; Tomlinson, L.; Blaney, F., A group program for obese, infertile women: weight loss and improved psychological health. *J Psychosom Obstet Gynaecol* **1996,** *17* (2), 125-8.
S95. Hollmann, M. R., B. Gerhard, I., Effects of weight loss on the hormonal profile in obese, infertile women. *Hum. Reprod.* **1996,** *11* (9), 1884-1891.
S96. Clark, A. M. L., W. Galletly, C. Tomlinson, L. Blaney, F. Wang, X. Norman, R. J., Weight loss results in significant improvement in pregnancy and ovulation rates in anovulatory obese women. *Hum. Reprod.* **1995,** *10* (10), 2705-2712.
S97. Munir, S. S.; Jabeen, S.; Alvi, N.; Khan, D.; Younis, B. B.; Khan, M., Role of orlistat combined with life style in the management of obese patients with polycystic ovarian syndrome. *PJMHS* **2018,** *12* (2), 477-480.
S98. Den Harink, T. G., R. B. J. B. Hoek, A. Groen, H. Blom, N. A. Roseboom, T. J. Van Deutekom, A. W. In *Preconception lifestyle intervention in obese women improves echocardiographic indices of cardiovascular function in their offspring: Follow up of a randomised controlled trial*, 53rd Annual Meeting of the Association for European Paediatric and Congenital Cardiology (AEPC) Seville,Spain, May15-18,2019; Cardiol Young: Seville,Spain, 2019; pp S11-S12.
S99. Van Elten, T. M.; Karsten, M. D. A.; Geelen, A.; Gemke, R.; Groen, H.; Hoek, A.; Van Poppel, M. N. M.; Roseboom, T. J., Preconception lifestyle intervention reduces long term energy intake in women with obesity and infertility: a randomised controlled trial *Int J Behav Nutr Phys Act* **2019,** *16* (1).
S100. Wekker, V.; Karsten, M. D. A.; Painter, R. C.; Van De Beek, C.; Groen, H.; Mol, B. W. J.; Hoek, A.; Laan, E.; Roseboom, T. J., Sexual function in obese infertile women 5-6 years after a preconception lifestyle intervention. *Int J Gynaecol Obstet* **2018,** *143*, 402-.

S101. van Elten, T. M.; Karsten, M. D. A.; Geelen, A.; van Oers, A. M.; van Poppel, M. N. M.; Groen, H.; Gemke, R.; Mol, B. W.; Mutsaerts, M. A. Q.; Roseboom, T. J.; et al., Effects of a preconception lifestyle intervention in obese infertile women on diet and physical activity; A secondary analysis of a randomized controlled trial. *PLoS ONE* **2018,** *13* (11), e0206888.
S102. Einarsson, S.; Christina, B.; Kluge, L.; Thurin-Kjellberg, A. In *The effect of weight intervention on obstetric and neonatal outcome in obese women scheduled for IVF treatment*, he 34rd Annual Meeting of the European Society of Human Reproduction and Embryology, July,2018; Hum. Reprod.: 2018; pp i319-i320.
S103. Wekker, V.; Van Dammen, L.; Van Oers, A. M.; Mutsaerts, M. A. Q.; Painter, R. C.; Zwinderman, A. H.; Groen, H.; Van De Beek, C.; Kobold, A. C. M.; Land, J. A.; et al. In *Effect of a lifestyle intervention in obese infertile women on cardiometabolic health and quality of life: results of a randomised controlled trial*, 33rd Annual Meeting of the European Society of Human Reproduction and Embryology, Geneva. Switzerland, July,2017; Hum. Reprod.: Geneva. Switzerland, 2017; pp i28-i29.
S104. Dammen, L. W., V.; Oers, A.; Mutsaerts, M.; Painter, R.;; Zwinderman, A. G., H.; Beek, C.; Kobold, A.; Land, J.;; Kuchenbecker, W. G., R.; Oosterhuis, G.; Vogel, N.; Mol, B.;Roseboom, T.; Group, A. In *Effect of a lifestyle intervention in obese infertile women on cardiometabolic health and quality of life*, 24th EuropeanCongress of the European Congress on Obesity(ECO2017), Porto,Portugal, May. 2017; Obes Facts: Porto,Portugal, 2017; pp p 3-4.
S105. Arentz, S.; Smith, C.; Abbott, J.; Fahey, P.; Cheema, B.; Bensoussan, A. In *Randomized controlled trial of combined lifestyle and herbal medicine in women with polycystic ovary syndrome*, 33rd Annual Meeting of the European Society of Human Reproduction and Embryology, Geneva. Switzerland, July.2017; Hum. Reprod.: Geneva. Switzerland, 2017; pp i31-i32.
S106. Van Oers, A. G., H. Mutsaerts, M. Burggraaff, J. Kuchenbecker, W. Perquin, D. Koks, C. Van Golde, R. Kaaijk, E. Schierbeek, J. Verberg, M. Broekmans, F. Mol, B. W. Land, J. Hoek, A. In *Is there a different effect of lifestyle intervention in subgroups of infertile obese women? Prespecified subgroup analyses of the LIFEstyle randomised controlled trial*, the 32nd Annual Meeting of the European Society of Human Reproduction and Embryology, Helsinki,Filand, July,2016; Hum. Reprod.: Helsinki,Filand, 2016; p i29.
S107. Legro, R. S.; Dodson, W. C.; Kunselman, A. R.; Stetter, C. M.; Kris-Etherton, P. M.; Williams, N. I.; Gnatuk, C. L.; Estes, S. J.; Allison, K. C.; Sarwer, D. B.; et al., Benefit of Delayed Fertility Therapy With Preconception Weight Loss Over Immediate Therapy in Obese Women With PCOS. *J. Clin. Endocrinol. Metab.* **2016,** *101* (7), 2658-2666.
S108. Legro, R. S. D., W. C. Kris-Etherton, P. M. Kunselman, A. R. Stetter, C. M. Williams, N. I. Gnatuk, C. L. Estes, S. J. Fleming, J. Allison, K. C. Sarwer, D. B. Coutifaris, C. Dokras, A., Randomized Controlled Trial of Preconception Interventions in Infertile Women With Polycystic Ovary Syndrome. *J. Clin. Endocrinol. Metab.* **2015,** *100* (11), 4048-4058.
S109. Duval, K.; Belan, M.; Jean-Denis, F.; Carranza-Mamane, B.; Pesant, M.-H.; Langlois, M.-F.; Hivert, M.-F.; Lavoie, H. B.; Baillargeon, J.-P., An Interdisciplinary Lifestyle Intervention Improves Clinically Relevant Fertility Outcomes in Obese Infertile Women – Preliminary Results of a Randomized Controlled Trial. *Can J Diabetes* **2015,** *39* (6), 532-533.
S110. Belan, M.; Duval, K.; Jean-Denis, F.; Pesant, M. H.; Langlois, M. F.; Ainmelk, Y.; Carranza-Mamane, B.; Baillargeon, J. P. In *Impacts of lifestyle and anthropometric changes in male partners of obese infertile women on couples' fertility-preliminary results from a cohort study*, 97th Annual Meeting and Expo of the Endocrine Society, (ENDO 2015), San Diego, CA. United States., March, 2015; Endocr. Rev.: San Diego, CA. United States., 2015.
S111. Van Oers, A. M.; Mutsaerts, M. A. Q.; Burggraaff, J. M.; Kuchenbecker, W. K. H.; Perquin, D. A. M.; Koks, C. A. M.; Van Golde, R.; Kaaijk, E. M.; Schierbeek, J. M.; Oosterhuis, G. J. E.; et al. In *Cost-effectiveness of a structured lifestyle program in overweight and obese subfertile women. Preliminary data from a randomised controlled trial-the LIFEstyle study*, 30th ESHRE Annual Meeting, Munich, Germany, July,2014; Hum. Reprod.: Munich, Germany, 2014; pp i354-i355.
S112. Mutsaerts, M. A. Q.; Van Oers, A. M.; Groen, H.; Burggraaff, J. M.; Kuchenbecker, W. K. H.; Perquin, D. A. M.; Koks, C. A. M.; Van Golde, R.; Kaaijk, E. M.; Schierbeek, J. M.; et al. In *The effectiveness of a structured lifestyle program in overweight and obese subfertile women. Preliminary data from a randomised controlled trial (LIFEstyle study)*, 30th ESHRE Annual Meeting, Munich, Germany, July,2014; Hum. Reprod.: Munich, Germany, 2014; pp i75-i76.
S113. Legro, R. S.; Dodson, W. C.; Kunselman, A. R.; Kris-Etherton, P. M.; Allison, K. C.; Sarwer, D. B.; Dokras, A.; Coutifaris, C. In *Effects of preconception intervention on the PCOS phenotype, ovulation, and live birth rates: a multicenter, multi-phase RCT*, 70th Annual Meeting of the American Society for Reproductive Medicine, Honolulu, Hawaii, October 2014; Fertil. Steril.: Honolulu, Hawaii, 2014; p e2.

S114. Sim, K. A. D., G. M. Denyer, G. S. Caterson, I. D. In *Substantial cost savings of a weight-loss programme in obese women undergoing fertility treatment*, 20th European Congress on Obesity, ECO 2013, Liverpool, United Kingdom, May,2013; Obes Facts: Liverpool, United Kingdom, 2013; p 46.
S115. Jakubowicz, D. B., M. Wainstein, J. Froy, O. In *Influence of meal timing on glucose metabolism, ovarian cytochrome P450C17α activity and serum free testosterone in lean women with polycystic ovary syndrome*, 95th Annual Meeting and Expo of the Endocrine Society, (ENDO 2013), San Francisco, CA, United States, June,2013; Endocr. Rev.: San Francisco, CA, United States, 2013.
S116. J., E.-G. J. Effect of Hypocaloric Diet and Exercise in Obese Women Who Are Subjected to IVF Cycle. https://www.cochranelibrary.com/central/doi/10.1002/central/CN-01490368/full (accessed 20.Sept.).
S117. Sim, K. D., G. Caterson, I., Weight loss improves reproductive outcomes for obese women undergoing assisted reproductive technology. *Obesity Facts* **2012,** *5* (suppl 1), 36.
S118. A.T., K. Weight Management Intervention for Obese Women and the Live Birth Outcome of in Vitro Fertilization (IVF). https://www.cochranelibrary.com/central/doi/10.1002/central/CN-01536498/full (accessed 20.Sept.).
S119. Ma, H., Zheng, Y.H. Evaluate the efficacy of abdominal acupuncture as a treatment for Obesity-type Poly cystic Ovarian Syndrome Patients: a randomized controlled trial. https://www.cochranelibrary.com/central/doi/10.1002/central/CN-01832945/full (accessed 20 Sept.).
S120. Palomba, S.; Falbo, A.; Giallauria, F.; Russo, T.; Rocca, M.; Tolino, A.; Zullo, F.; Orio, F., Six weeks of structured exercise training and hypocaloric diet increases the probability of ovulation after clomiphene citrate in overweight and obese patients with polycystic ovary syndrome: a randomized controlled trial. *Hum. Reprod.* **2010,** *25* (11), 2783-2791.
S121. Yin, Y.; Zhang, Y.; Zhang, H.; Jiang, D.; Guo, G., Clinical therapeutic effects of acupuncture combined with Chinese herbal medicine on infertility of polycystic ovary syndrome in the patients with ovulation induction with letrozole. *Zhongguo zhen jiu* **2018,** *38* (1), 27-32.
S122. Shen, L. Y.; Liang, C. M.; Yang, W. J.; Pan, L.; Li, H.; Hu, H., Acupuncture Treatment of Polycystic Ovarian Syndrome Patients with Abdominal Obesity by Regulating Dai Meridian: a Randomized Controlled Clinical Trial. *Zhen Ci Yan Jiu* **2018,** *43* (4), 255-259.
S123. Cao, Y.; Zhang, L.; Zhao, D.; Liu, Z., DONG's extraordinary acupoints for the ovarian function of polycystic ovary syndrome: a randomized controlled pilot trial. *Zhongguo Zhen Jiu* **2017,** *37* (7), 710-714.
S124. Nasrekani, Z. A.; Fathi, M., Efficacy of 12 weeks aerobic training on body composition, aerobic power and some women-hormones in polycystic ovary syndrome infertile women. *Iranian journal of obstetrics, gynecology and infertility* **2016,** *19* (5), 1-10.
S125. Lai, M. H. M., H. X. Yao, H. Liu, H. Song, X. H. Huang, W. Y. Wu, X. K., Effect of abdominal acupuncture therapy on the endocrine and metabolism in obesity-type polycystic ovarian syndrome patients. *Zhen Ci Yan Jiu* **2010,** *35* (4), 298-302.
S126. Hamayeli Mehrabani, H. T., F. Salehpour, S. Hedayati, M. Amiri, Z. Ghassemi, A., Reproductive hormonal changes following two types of hypocaloric diets in overweight and obese polycystic ovary syndrome women. *IJEM* **2010,** *12* (2), 160-168+200.
S127. Esmaeilzadeh, S. G., L. Sharbatdaran, M. Bijani, A. Sajadi, P., Comparison of flutamide and metformin in overweight-obese women with polycystic ovary syndrome following a hypocaloric dieting. *JBUMS* **2010,** *12* (4), 7-13.
S128. J.P., B. Fit-for-Fertility Multicenter Randomized Controlled Trial. https://www.cochranelibrary.com/central/doi/10.1002/central/CN-01919678/full (accessed Sep).
S129. G., C. Multidisciplinary Treatment of Obesity Prior to in Vitro Fertilization: impact on Global Reproductive Outcomes (PRO-FIV Study). https://www.cochranelibrary.com/central/doi/10.1002/central/CN-01559547/full (accessed Sep.).
S130. T., S. A trial to compare the effect of weight loss diets on obese women with polycystic ovarian syndrome (PCOS). https://www.cochranelibrary.com/central/doi/10.1002/central/CN-01895098/full (accessed Sep.).
S131. Jiskoot, G.; Benneheij, S. H.; Beerthuizen, A.; De Niet, J. E.; De Klerk, C.; Timman, R.; Busschbach, J. J.; Laven, J. S. E., A three-component cognitive behavioural lifestyle program for preconceptional weight-loss in women with polycystic ovary syndrome (PCOS): a protocol for a randomized controlled trial. *Reprod Health* **2017,** *14* (34).
S132. Duval, K.; Langlois, M.-F.; Carranza-Mamane, B.; Pesant, M.-H.; Hivert, M.-F.; Poder, T. G.; Lavoie, H. B.; Ainmelk, Y.; Tribble, D. S.-C.; Laredo, S. J. B. o., The Obesity-Fertility Protocol: a randomized controlled trial assessing clinical outcomes and costs of a transferable interdisciplinary lifestyle intervention, before and during pregnancy, in obese infertile women. *BMC obesity* **2015,** *2* (1), 47.

S133. Santoro N., Z. H., Legro R., Diamond M.,Cedars M., Steiner A., Hansen K., Coutifaris C.and Eisenberg E. Improving Reproductive Fitness Through Pretreatment With Lifestyle Modification in Obese Women With Unexplained Infertility. https://www.cochranelibrary.com/central/doi/10.1002/central/CN-01506150/full (accessed Sep).
S134. Mutsaerts, M. A.; Groen, H.; ter Bogt, N. C.; Bolster, J. H.; Land, J. A.; Bemelmans, W. J.; Kuchenbecker, W. K.; Hompes, P. G.; Macklon, N. S.; Stolk, R. P.; et al., The LIFESTYLE study: costs and effects of a structured lifestyle program in overweight and obese subfertile women to reduce the need for fertility treatment and improve reproductive outcome. A randomised controlled trial. *BMC Womens Health* **2010,** *10*, 22.
S135. van Elten, T. M.; Karsten, M. D. A.; Geelen, A.; Gemke, R. J. B. J.; Groen, H.; Hoek, A.; van Poppel, M. N. M.; Roseboom, T. J., Preconception lifestyle intervention reduces long term energy intake in women with obesity and infertility: a randomised controlled trial. *IInt J Behav Nutr Phys Act* **2019,** *16* (1), N.PAG-N.PAG.
S136. van Dammen, L.; Wekker, V.; de Rooij, S. R.; Mol, B. W. J.; Groen, H.; Hoek, A.; Roseboom, T. J., The effects of a pre-conception lifestyle intervention in women with obesity and infertility on perceived stress, mood symptoms, sleep and quality of life. *PLoS ONE* **2019,** *14* (2).
S137. Einarsson, S.; Bergh, C.; Kluge, L.; Thurin-Kjellberg, A., No effect of weight intervention on perinatal outcomes in obese women scheduled for in vitro fertilization treatment. *Acta Obstet Gynecol Scand* **2019**.
S138. van Elten, T. M. K., M. D. A. Geelen, A. van Oers, A. M. van Poppel, M. N. M. Groen, H. Gemke, Rjbj Mol, B. Mutsaerts, M. A. Q. Roseboom, T. J. Hoek, A. L. IFEstyle Study Grp, Effects of a preconception lifestyle intervention in obese infertile women on diet and physical activity; A secondary analysis of a randomized controlled trial. *PloS ONE* **2018,** *13* (11), 15.
S139. Wekker, V.; Karsten, M. D. A.; Painter, R. C.; van de Beek, C.; Groen, H.; Mol, B. W. J.; Hoek, A.; Laan, E.; Roseboom, T. J., A lifestyle intervention improves sexual function of women with obesity and infertility: a 5 year follow-up of a RCT. *PloS ONE* **2018,** *13* (10), e0205934.
S140. van Dammen, L.; Wekker, V.; Van Oers, A. M.; Mutsaerts, M. A. Q.; Painter, R. C.; Zwinderman, A. H.; Groen, H.; van de Beek, C.; Muller Kobold, A. C.; Kuchenbecker, W. K. H.; et al., Effect of a lifestyle intervention in obese infertile women on cardiometabolic health and quality of life: a randomized controlled trial. *PloS ONE* **2018,** *13* (1) (no pagination).
S141. Van Oers, A. M.; Mutsaerts, M. A. Q.; Burggraaff, J. M.; Kuchenbecker, W. K. H.; Perquin, D. A. M.; Koks, C. A. M.; Van Golde, R.; Kaaijk, E. M.; Schierbeek, J. M.; Klijn, N. F.; et al., Cost-effectiveness analysis of lifestyle intervention in obese infertile women. *Hum. Reprod.* **2017,** *32* (7), 1418-1426.
S142. van Oers, A. M.; Groen, H.; Mutsaerts, M. A.; Burggraaff, J. M.; Kuchenbecker, W. K.; Perquin, D. A.; Koks, C. A.; van Golde, R.; Kaaijk, E. M.; Schierbeek, J. M.; et al., Effectiveness of lifestyle intervention in subgroups of obese infertile women: a subgroup analysis of a RCT. *Hum. Reprod.* **2016,** *31* (12), 2704-2713.
S143. Moran, L. J.; Tsagareli, V.; Noakes, M.; Norman, R., Altered Preconception Fatty Acid Intake Is Associated with Improved Pregnancy Rates in Overweight and Obese Women Undertaking in Vitro Fertilisation. *Nutrients* **2016,** *8* (1).
S144. Jiskoot, G.; Timman, R.; Beerthuizen, A.; Dietz de Loos, A.; van Busschbach, J.; Laven, J., Results Of A Lifestyle Intervention Involving Healthy Diet, Exercise and Cognitive Behavioral Therapy In Polycystic Ovary Syndrome (PCOS). *Am. J. Physiol. Endocrinol. Metab.* **2018**.
S145. Belan, M.; Carranza-Mamane, B.; Ainmelk, Y.; Pesant, M.-H.; Duval, K.; Jean-Denis, F.; Langlois, M.-F.; Lavoie, H. B.; Waddell, G.; Baillargeon, J.-P. J. C. J. o. D., A Lifestyle Intervention Targeting Women With Obesity and Infertility Improves Their Fertility Outcomes, Especially in Women With PCOS: A Randomized Controlled Trial. *Can J Diabetes* **2019,** *43* (7), S2.
S146. Belan, M.; Carranza-Mamane, B.; AinMelk, Y.; Pesant, M.-H.; Duval, K.; Jean-Denis, F.; Langlois, M.-F.; Lavoie, H.; Waddell, G.; Baillargeon, J.-P. J. F.; Sterility, A lifestyle intervention targeting women with obesity and infertility improves their fertility outcomes, especially in women with PCOS: a randomized controlled trial. *Fertil. Steril.* **2019,** *112* (3), e40.
S147. Duval, K. B., M. Jean-Denis, F. Baillargeon, J. In *An interdisciplinary lifestyle intervention improves clinically relevant fertility outcomes in obese infertile women-preliminary results*, 71st Annual Meeting of the American Society for Reproductive Medicine, (ASRM 2015), Oct,2015; Fertil. Steril.: 2015; p e97.

© 2020 by the authors. Submitted for possible open access publication under the terms and conditions of the Creative Commons Attribution (CC BY) license (http://creativecommons.org/licenses/by/4.0/).
