# Supplementary material for: Effectiveness of Non-Pharmacological Interventions for Overweight or Obese Infertile Women: A Systematic Review and Meta-Analysis
Source: Int J Environ Res Public Health. 2020 Oct 13;17(20):7438. doi: 10.3390/ijerph17207438 (PMC7650570; doi:10.3390/ijerph17207438)
Supplement: Supplementary file 1 [file ijerph-17-07438-s001.zip › supplementary file_S3.pdf]

International Journal of
Environmental Research and Public Health

MDPI

Supplementary File S3: Sensitivity analysis

Study or Subgroup	Experimental Events	Experimental Total	Control Events	Control Total	Weight	Risk Ratio M-H, Random, 95% CI
Becker 2015	3	14	0	12	0.6%	6.07 [0.34, 106.85]
Belan 2019	31	51	22	57	27.9%	1.57 [1.06, 2.34]
Einarsson 2017	53	152	47	153	40.1%	1.14 [0.82, 1.57]
Espinos 2017	12	21	7	20	9.4%	1.63 [0.81, 3.30]
Moran 2011	12	18	8	20	11.7%	1.67 [0.89, 3.12]
Mutsaerts 2016	175	280	186	284	0.0%	0.95 [0.84, 1.08]
Qublan 2007	8	24	6	22	6.0%	1.22 [0.50, 2.97]
Rothberg 2016	3	6	0	5	0.6%	6.00 [0.38, 94.35]
Sim 2014	13	27	3	22	3.7%	3.53 [1.15, 10.84]
**Total (95% CI)**		**313**		**311**	**100.0%**	**1.44 [1.16, 1.79]**
Total events	135		93			

Heterogeneity: Tau² = 0.00; Chi² = 7.25, df = 7 (P = 0.40); I² = 3%
Test for overall effect: Z = 3.27 (P = 0.001)

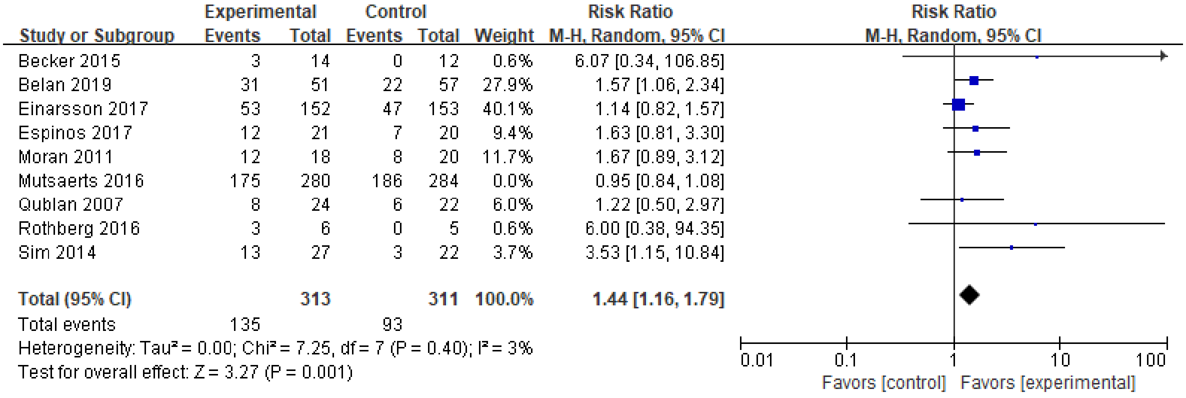

Figure S1. Post hoc sensitivity analysis-pregnancy rate.

Study or Subgroup	Experimental Events	Experimental Total	Control Events	Control Total	Weight	Risk Ratio M-H, Random, 95% CI
Becker 2015	3	14	0	12	1.1%	6.07 [0.34, 106.85]
Belan 2019	26	51	21	57	30.3%	1.38 [0.90, 2.13]
Einarsson 2017	45	152	42	153	37.7%	1.08 [0.76, 1.54]
Espinos 2017	13	21	6	20	13.8%	2.06 [0.98, 4.36]
Moran 2011	7	18	5	20	9.1%	1.56 [0.60, 4.04]
Mutsaerts 2016	123	280	153	284		Not estimable
Rothberg 2016	3	6	0	5	1.2%	6.00 [0.38, 94.35]
Sim 2014	12	27	3	22	6.7%	3.26 [1.05, 10.12]
**Total (95% CI)**		**289**		**289**	**100.0%**	**1.48 [1.08, 2.01]**
Total events	109		77			

Heterogeneity: Tau² = 0.03; Chi² = 7.43, df = 6 (P = 0.28); I² = 19%
Test for overall effect: Z = 2.48 (P = 0.01)

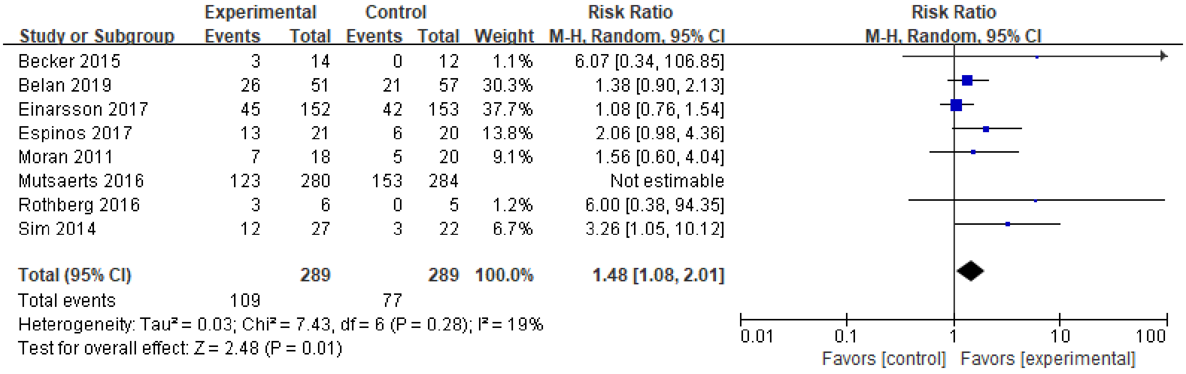

Figure S1. Post hoc sensitivity analysis-live birth rate.

Study or Subgroup	Experimental Events	Experimental Total	Control Events	Control Total	Weight	Risk Ratio M-H, Fixed, 95% CI
Einarsson 2017	8	152	5	153	61.1%	1.61 [0.54, 4.81]
Espinos 2017	1	21	1	20	12.6%	0.95 [0.06, 14.22]
Mutsaerts 2016	41	280	27	284	0.0%	1.54 [0.98, 2.43]
Qublan 2007	1	24	1	22	12.8%	0.92 [0.06, 13.79]
Sim 2014	5	27	1	22	13.5%	4.07 [0.51, 32.35]
**Total (95% CI)**		**224**		**217**	**100.0%**	**1.77 [0.76, 4.12]**
Total events	15		8			

Heterogeneity: Chi² = 1.08, df = 3 (P = 0.78); I² = 0%
Test for overall effect: Z = 1.33 (P = 0.18)

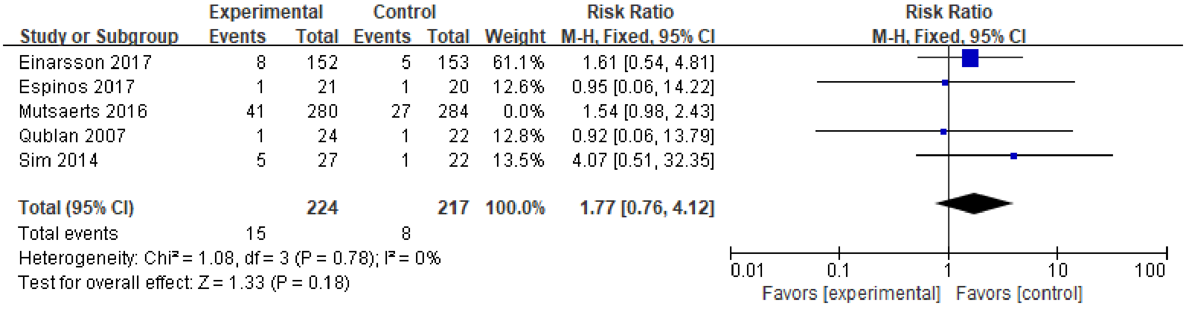

Figure S2. Post hoc sensitivity analysis--miscarriage rate.

© 2020 by the authors. Submitted for possible open access publication under the terms and conditions of the Creative Commons Attribution (CC BY) license (http://creativecommons.org/licenses/by/4.0/).
